# Gut Microbiome in Sporadic Cerebral Small Vessel Disease: MRI-Defined Phenotypes, Mechanistic Evidence, and Nutritional Implications for Brain Aging

**DOI:** 10.3390/nu18203421

**Published:** 2026-10-18

**Authors:** Tamas Jarecsny, Ferenc Fazekas, Nadim Al-Muhanna, Richard Schwab, Viktoria Hadnagy, Sara Laki, Janos Tamas Varga, Andrea Lehoczki, Virag Zabo, Diana Gorog, Monika Fekete

**Affiliations:** 1 Department of Neurology and Stroke, Saint John’s Central Hospital of North Buda, 1125 Budapest, Hungary; jarecsny.tamas@janoskorhaz.hu (T.J.); fazekas.ferenc@janoskorhaz.hu (F.F.); nadim@janoskorhaz.hu (N.A.-M.); 2 Health Sciences Division, Doctoral College, Semmelweis University, 1085 Budapest, Hungary; lehoczki.andrea.marianna@semmelweis.hu (A.L.); zabo.virag@semmelweis.hu (V.Z.); 3 MiND Brain-Gut Center, 1125 Budapest, Hungary; mailbox@schwab.hu (R.S.); viktoria.hadnagy@biomteam.hu (V.H.); sara.laki@biomteam.hu (S.L.); 4 Department of Pulmonology, Semmelweis University, 1083 Budapest, Hungary; varga.janos.tamas@semmelweis.hu; 5 Institute of Preventive Medicine and Public Health, Faculty of Medicine, Semmelweis University, 1089 Budapest, Hungary; 6 Fodor Center for Prevention and Healthy Aging, Semmelweis University, 1085 Budapest, Hungary; 7 Faculty of International Business Economics, Budapest University of Economics and Business, 1054 Budapest, Hungary; gorogdia2004@gmail.com

**Keywords:** cerebral small vessel disease, gut microbiome, brain aging, white matter hyperintensities, magnetic resonance imaging, vascular cognitive impairment, gut–brain axis, microbial richness

## Abstract

Background/Objectives: Cerebral small vessel disease (CSVD) is a major vascular component of brain aging, yet potentially modifiable systemic contributors remain incompletely understood. Growing evidence links the gut microbiome to magnetic resonance imaging (MRI)-defined CSVD, but interpretation is complicated by heterogeneity in microbiome profiling, clinical populations, analytical methods, and neuroimaging phenotypes. This review critically integrates the available human evidence with complementary multi-omics, ex vivo, and experimental findings. Methods: PubMed, Embase, Scopus, and Web of Science were searched through 4 August 2026; 12 eligible reports representing eight cohort families were included and evaluated using a structured study-level methodological appraisal. Results: Across the available literature, ecological and host-related biological processes show greater qualitative convergence than individual taxa, although this pattern has not been formally quantified across independent cohorts. Among reported alpha diversity measures, Chao/Chao1 showed the same numerical disease-versus-control direction across four cohort families, with statistical significance in two, suggesting a recurrent but still preliminary richness-related signal; Shannon and Simpson indices were less consistent. No single genus or species has been consistently replicated across the available, largely cross-sectional cohorts. Complementary studies implicate intestinal barrier, immune–metabolic, neurovascular, and perivascular clearance pathways but do not establish a unified causal pathway in human sporadic CSVD. Conclusions: These observations support a hypothesis-generating ecosystem-level framework. Because diet is an important modifiable determinant of the gut microbial ecosystem, these findings may have relevance for nutrition-based strategies targeting vascular brain aging. However, no CSVD-specific dietary or microbiome-directed intervention has yet been shown to modify disease progression or related MRI outcomes.

## 1. Introduction

Population aging is increasing the global burden of neurological and cognitive disorders [1,2]. The number of people aged 60 years or older is projected to reach approximately 1.4 billion by 2030 and 2.1 billion by 2050 [3]. Disorders of the nervous system affected an estimated 3.4 billion people in 2021 and were the leading group cause of disability-adjusted life-years worldwide [4]. Dementia affected approximately 57 million people in 2021 and carries substantial health, social care, and economic costs [5]. Identifying modifiable contributors to brain aging is therefore an important public health priority.

Cerebral small vessel disease (CSVD) is among the most prevalent vascular pathologies affecting the aging brain. Magnetic resonance imaging (MRI) manifestations include white matter hyperintensities (WMHs), lacunes, cerebral microbleeds (CMBs), enlarged perivascular spaces (EPVSs), recent small subcortical infarcts, and diffuse white matter injury [6,7]. Sporadic CSVD is primarily a cerebrovascular disorder rather than a primary neurodegenerative disease, and it contributes to stroke, gait impairment, vascular cognitive impairment and dementia, and loss of independence [6,7,8]. Greater WMH burden is associated with increased risks of future stroke, dementia, and mortality [9,10].

Vascular and neurodegenerative changes frequently coexist in older adults. Small vessel injury may reduce cerebral reserve and increase the clinical impact of Alzheimer-type pathology [11]. Endothelial dysfunction, blood–brain barrier disruption, inflammation, impaired perfusion, and altered perivascular clearance are relevant to both vascular cognitive impairment and broader brain aging [7,8,12,13]. These shared pathways have increased interest in systemic modifiers of vascular vulnerability [14].

The gut microbiome is one such candidate. Human interventions demonstrate that diet and fermented food exposure can modify microbial composition, function, and systemic immune profiles [15]. Gut microbial communities influence nutrient metabolism, immune regulation, intestinal barrier integrity, and the production of systemic metabolites; microbiota–gut–brain communication can occur through immune, metabolic, endocrine, and neural pathways, and microbial products may affect endothelial and blood–brain barrier function [14,16,17]. More broadly, dietary patterns, prebiotics, probiotics, fermented foods, and other microbiome-directed approaches provide testable strategies for modifying the microbial ecosystem, although their relevance to CSVD has not been established [15,16,18,19].

Previous reviews have addressed broader cerebrovascular disease, microbiota–gut–brain mechanisms in CSVD, or exercise-related microbiota–short-chain-fatty-acid–inflammation pathways [16,17,20]. However, human evidence centered specifically on stool-derived microbiome profiles and MRI-defined sporadic CSVD remains difficult to interpret because study designs, microbial methods, imaging phenotypes, and cohort independence vary substantially.

Human studies directly linking the gut microbiome to CSVD have increased in recent years. Early reports focused on enterotypes and WMHs; later studies incorporated 16S ribosomal RNA (rRNA) sequencing, shotgun metagenomics, metabolomics, quantitative MRI, neurovascular coupling measures, and glymphatic proxies. Interpretation is difficult because studies differ in CSVD definition, imaging phenotype, sequencing method, diversity metric, taxonomic resolution, confounder adjustment, and multiple-testing control. Several reports also arise from overlapping samples, so publication counts may overestimate independent evidence [21,22,23,24,25,26,27,28,29,30,31,32].

This review critically integrates human studies of the gut microbiome in sporadic CSVD, with emphasis on MRI-defined phenotypes, cognition, and mechanisms relevant to brain aging. Rather than seeking a single disease-associated taxon, we examine evidence across community structure, phenotype-specific taxonomic associations, conventional and advanced neuroimaging, and complementary mechanistic and multi-omics findings. We also consider how methodological fragmentation shapes cross-study comparability and whether recurring ecological and functional patterns identify potentially modifiable research targets relevant to nutrition and brain aging.

## 2. Literature Search and Review Approach

### 2.1. Scope of the Review

This review examined human evidence linking stool-derived gut microbiome characteristics with MRI-defined sporadic CSVD and its individual neuroimaging phenotypes. Particular attention was given to microbial community structure, alpha and beta diversity, taxonomic associations, conventional and advanced neuroimaging findings, cognition, and complementary biological evidence relevant to the gut–vascular–brain axis.

The review focused primarily on human observational evidence. Metabolomic, inflammatory, intestinal barrier, ex vivo, and experimental findings were additionally considered when they contributed to interpretation of mechanisms potentially linking the gut microbial ecosystem with cerebral small vessel injury. Because several publications originated from overlapping or potentially overlapping participant samples, cohort relationships were considered when evaluating the recurrence and independence of reported findings. This review was designed as a structured critical narrative review rather than a systematic or scoping review. Structured database searching, transparent study selection, cohort overlap assessment, and study-level methodological appraisal were used to improve reproducibility, while the primary objective was the critical integration of heterogeneous observational, multi-omics, ex vivo, and experimental evidence rather than protocol-driven systematic evidence grading or quantitative synthesis.

### 2.2. Literature Search

To inform this review, searches were conducted in PubMed, Embase via Embase.com, Scopus, and Web of Science Core Collection from database inception to 4 August 2026. Searches combined controlled vocabulary, where available, with title, abstract, and keyword terms related to the gastrointestinal or stool microbiome and CSVD or its neuroimaging manifestations.

Search terms covered gut microbiome/microbiota, fecal/faecal microbiome, dysbiosis, metagenomics, 16S rRNA, cerebral small vessel disease, cerebral microangiopathy, white matter hyperintensities, leukoaraiosis, Fazekas, lacunes, cerebral microbleeds, and perivascular spaces. No date or language restrictions were applied. A mandatory MRI search block and broad Boolean exclusion operators were avoided to preserve sensitivity because relevant studies could describe individual CSVD manifestations without explicitly using MRI or CSVD terminology.

The PubMed strategy was adapted to the syntax and controlled vocabulary of the other databases. Database-specific strategies, platforms, search dates, and search details are provided in Supplementary Table S1. No separate systematic search of grey literature, preprint servers, or backward citations was performed. Conference abstracts identified through the database searches were reviewed, and where relevant, we checked whether a corresponding full-text publication had subsequently become available.

### 2.3. Identification of Relevant Evidence

Eligible studies were required to meet all of the following criteria: (1) original quantitative human research involving adults; (2) stool-derived multi-taxon gut microbiome profiling; (3) cerebral MRI assessment; and (4) interpretable information on the relationship between microbiome characteristics and sporadic CSVD, an individual CSVD imaging phenotype, or a closely related quantitative imaging measure.

Relevant microbiome approaches included 16S rRNA gene sequencing, shotgun metagenomics, terminal restriction fragment length polymorphism, and other documented multi-taxon profiling techniques. Microbiome features of interest included alpha and beta diversity, taxonomic abundance, enterotypes, community patterns, and sequencing-derived functional profiles.

The principal disease context was sporadic CSVD, predominantly associated with aging and vascular risk. Studies centered on monogenic small vessel disorders—including cerebral autosomal dominant arteriopathy with subcortical infarcts and leukoencephalopathy, cerebral autosomal recessive arteriopathy with subcortical infarcts and leukoencephalopathy, other HTRA1-related disorders, Fabry disease, primary cerebral amyloid angiopathy, and other clearly secondary small vessel disorders—were not considered part of the core evidence unless data relevant to sporadic CSVD could be separately interpreted.

Previous or acute stroke did not automatically preclude consideration of a study, but stroke itself was not treated as an outcome of interest. Studies involving stroke populations were considered only when chronic CSVD imaging features were objectively assessed and the microbiome–CSVD relationship could be distinguished from the acute cerebrovascular event. Single-taxon assays, culture-only studies, case reports, non-analytical case series, reviews, editorials, protocols, and studies without direct stool microbiome profiling were not included in the core human evidence.

Search results were deduplicated using exact DOI, PMID, and normalized title. Of 660 retrieved records, 234 duplicates were removed, and 426 unique records underwent title/abstract screening. Title/abstract screening and full-text eligibility assessment were performed by both T.J. and M.F. Disagreements were resolved by consensus. Twenty-seven reports underwent full-text eligibility assessment, and 12 were included. The complete selection flow, including the main exclusion reasons and related or superseded reports, is provided in Supplementary Figure S1 and Table S2.

### 2.4. Identification of Related and Overlapping Cohorts

Particular attention was given to publications that may have originated from the same or overlapping participant populations. Cohort relationships were evaluated using recruitment center and period, sample size, participant characteristics, group composition, authorship, eligibility criteria and ethics information. Cohorts were treated as distinct when available recruitment information provided no evidence of shared participants; overlap was classified as probable when several concordant features strongly suggested shared recruitment and as potential when overlap could not be excluded but available information was insufficient for stronger classification. The term “provisionally independent” indicates that no evidence of shared participants was identified in the available publications; it does not establish complete cohort independence. A structured summary of the evidence supporting each cohort overlap classification is provided in Supplementary Table S3.

Related publications were allowed to contribute complementary phenotypes or analyses, but findings derived from the same or potentially overlapping participant sample were not interpreted as independent replication of the same biological association. Where several related reports addressed similar outcomes, the most complete and appropriately adjusted analysis was given greater interpretive weight. Cohort relationships are summarized in Table 1.

### 2.5. Evidence Mapping and Interpretive Approach

Study-level data were extracted by T.J. and verified by M.F. Extracted variables included study setting and design, sample size and participant characteristics, recruitment details, microbiome methodology, neuroimaging phenotype, statistical approach, covariate adjustment, multiple-testing procedures, principal findings, and information relevant to potential cohort overlap. Any discrepancies identified during verification were resolved by discussion.

Microbiome findings were organized according to the biological dimension they represented. Community-level features included richness-focused measures such as Chao/Chao1 and the abundance-based coverage estimator, broader alpha diversity indices such as Shannon and Simpson, and beta diversity or community composition analyses. Taxonomic findings were considered separately and interpreted in relation to taxonomic rank, direction of association, MRI phenotype, statistical adjustment, correction for multiple comparisons, and cohort independence.

Neuroimaging findings were mapped according to conventional CSVD manifestations—including WMHs, lacunes, CMBs, EPVSs, and composite CSVD burden—as well as advanced imaging phenotypes related to white matter microstructure, venous abnormalities, neurovascular coupling, and MRI-derived perivascular clearance measures. Cognitive, metabolomic, inflammatory, intestinal barrier, and experimental findings were additionally considered when they contributed to interpretation of the gut–vascular–brain relationship. Human observational, ex vivo, and animal evidence was kept conceptually distinct.

Particular attention was given to methodological features capable of influencing cross-study comparability, including stool and sequencing methodology, bioinformatic pipelines, diversity metrics, taxonomic resolution, neuroimaging definitions, confounder adjustment, and multiple-testing procedures. These differences were considered not only as limitations of individual studies but also as potential contributors to the heterogeneity of the literature.

Interpretive weight was guided by cohort independence, sample size and statistical precision, degree of confounder adjustment, control of multiple testing, completeness of microbiome and neuroimaging methodology, and the directness of the reported microbiome–CSVD association.

The literature was interpreted through a critical narrative synthesis focused on recurring ecological patterns, phenotype-specific taxonomic associations, methodological sources of heterogeneity, cohort independence, and convergence of mechanistic findings across complementary levels of evidence.

### 2.6. Study-Level Methodological Appraisal

The included human studies were appraised using the Joanna Briggs Institute Critical Appraisal Tool for Analytical Cross-Sectional Studies. Ex vivo and animal experimental components were described separately and were not assigned cross-sectional appraisal judgments. The appraisal was used to identify study-level methodological limitations relevant to interpretation rather than to generate an overall numerical quality score or to determine study eligibility. Item-level judgments were based on the information reported in the primary publications and Supplementary Materials. Detailed appraisal results are provided in Supplementary Table S7.

## 3. Current Evidence Linking the Gut Microbiome to Cerebral Small Vessel Disease

### 3.1. Overview of the Available Evidence

The literature search identified 12 relevant reports published between 2019 and 2026, representing eight provisionally distinct cohort families after potential participant overlap was considered [21,22,23,24,25,26,27,28,29,30,31,32]. Nine reports originated from China, two from Japan, and one from the United States. Publication-level sample sizes ranged from 41 to 972 participants, with the Framingham Heart Study providing the largest population-based sample; most other studies were hospital-based or single-center.

All available human microbiome–CSVD analyses were cross-sectional. None prospectively examined whether baseline microbiome characteristics predicted subsequent progression of MRI-defined CSVD or cognitive decline. Study populations included CSVD-versus-control comparisons, memory clinic populations, cognitively defined subgroups, CSVD burden strata, and broader population samples in which microbiome features were related to continuous neuroimaging measures [21,22,23,24,25,26,27,28,29,30,31,32].

WMHs were the most frequently studied structural manifestation and were assessed using Fazekas grading, quantitative volume, or phenotype-based inclusion criteria. Other conventional outcomes included lacunes, CMBs, EPVSs, and composite CSVD burden scores. Several studies extended the phenotype to white matter microstructure, deep medullary veins, resting-state or neurovascular coupling measures, MRI-derived perivascular clearance proxies, and cognition [21,22,23,24,25,26,27,28,29,30,31,32].

Cohort overlap was an important feature of the evidence base. The two Japanese GIMLET reports originated from the same memory clinic recruitment framework [22,30]. Four Anhui publications were considered probably or potentially overlapping on the basis of recruitment setting, participant characteristics and authorship [24,25,27,31]. By contrast, the two Sun Yat-sen studies had non-overlapping recruitment periods and were treated as separate cohort families [26,29]. Framingham, Maoming, Wuxi, and Guangdong also represented distinct cohorts based on the available information [21,23,28,32]. Table 1 summarizes study characteristics, microbiome methodology, neuroimaging phenotypes, and cohort relationships. Additional study-level demographic, recruitment, exposure, and methodological characteristics are provided in Supplementary Table S4.

### 3.2. Methodological Heterogeneity Across Microbiome–CSVD Studies

The available studies differed substantially in how both the microbiome and CSVD were characterized. Nine reports used 16S rRNA gene amplicon sequencing, two GIMLET reports used terminal restriction fragment length polymorphism with enterotype-based analyses, and one study used shotgun metagenomics [21,22,23,24,25,26,27,28,29,30,31,32]. Among 16S studies, amplified regions included V4 and V3–V4, sequencing platforms included MiSeq and NovaSeq, and bioinformatic processing varied between operational taxonomic unit- and amplicon sequence variant-based approaches. Clustering thresholds, taxonomic databases, and differential abundance methods also differed, and human sequencing details were incompletely reported in the Guangdong study. Such methodological choices can materially affect microbiome profiling and differential abundance results [33,34].

Community-level ecological measures were similarly heterogeneous. Alpha diversity analyses included Chao/Chao1, the abundance-based coverage estimator, observed richness, Shannon, Simpson, Pielou evenness, and Faith phylogenetic diversity. Beta diversity and community composition analyses used Bray–Curtis, UniFrac, Jaccard, principal coordinate approaches, permutational multivariate analysis of variance, partial least-squares discriminant analysis, or related methods. Consequently, studies labeled broadly as assessing “diversity” frequently measured different ecological properties or used different statistical approaches to community separation.

Statistical treatment also varied considerably. Differential abundance analyses, correlations, regression models, receiver operating characteristic analyses, and machine learning approaches were used across studies. Some analyses applied false discovery rate or Bonferroni correction, whereas others relied predominantly on nominal or unadjusted associations. Adjustment for demographic and vascular covariates ranged from extensive multivariable modeling to unadjusted correlation matrices. An additional methodological consideration is the compositional nature of sequencing-derived microbiome data, in which relative abundances are constrained by the total observed community. Consequently, apparent increases or decreases in individual taxa may partly reflect changes in other community members rather than absolute changes in microbial load. Differential abundance findings derived from conventional relative abundance comparisons or correlation-based approaches should therefore be interpreted cautiously, particularly when compositionality is not explicitly addressed in the analytical method [33,34].

Neuroimaging methodology added a further layer of variation. MRI field strength ranged from 1.5 T to 3 T where reported. WMHs were characterized by Fazekas grading, volumetry, or both, while lesion counts and definitions varied for lacunes, CMBs, and EPVSs. Composite burden scores were also not uniform: some studies used a conventional 0–4 CSVD score, whereas the Anhui alpha diversity study used a modified 0–8 score that omitted EPVSs and weighted individual lesion types differently [29,31]. Advanced imaging studies additionally examined peak width of skeletonized mean diffusivity (PSMD), deep medullary veins, neurovascular coupling, resting-state measures, diffusion-derived indices, and MRI-based glymphatic or perivascular clearance proxies [21,23,24,25,27,28].

Several potentially important modifiers were inconsistently reported or adjusted for, including diet, medication and antibiotic exposure, vascular risk factors, sample handling, and the interval between stool collection and MRI. These methodological differences limit direct comparison across studies and require individual findings to be interpreted within the specific microbiome, statistical, clinical, and neuroimaging framework in which they were generated. Table 2 summarizes the principal sources of heterogeneity and their implications for interpretation.

### 3.3. Community-Level Microbiome Features

Alpha diversity findings differed according to both the ecological metric and the clinical contrast examined. The two GIMLET studies did not provide sequencing-derived Chao/Chao1 or Shannon estimates and were therefore not informative for direct comparison of these measures [22,30].

Across four disease-versus-control cohort families with an extractable Chao/Chao1 direction, richness was numerically lower in CSVD than in controls. In the Sun Yat-sen shotgun cohort, both Chao1 and Shannon indices were lower in arteriosclerotic CSVD (*p* = 0.0027 and *p* = 0.0053, respectively) [26]. In the preferred Anhui alpha diversity report, Chao1 was lower in CSVD (*p* < 0.001), whereas Shannon did not differ (*p* = 0.493 for ln-transformed values) [31]. The Wuxi cohort showed a lower Chao1 value that did not reach conventional statistical significance (*p* = 0.077), together with lower Shannon diversity (*p* = 0.033) [28]. In the small Guangdong CSVD-with-cognitive-impairment cohort, both Chao and Shannon were numerically lower than in controls but were not statistically significant [32]. Overall, this represents a directionally recurrent but statistically inconsistent richness pattern, with significant group differences in two of the four cohort families.

A potentially overlapping Anhui report provided additional within-family information: Chao1 and the abundance-based coverage estimator were lower in CSVD (*p* = 0.036 and *p* = 0.029), whereas Shannon and Simpson indices did not differ [27,31].

Other studies examined different clinical contrasts. Framingham found no significant relationship between global alpha diversity measures and continuous CSVD-related neuroimaging burden [21]. In contrast, Cai et al. reported higher Chao1, observed operational taxonomic units, and Faith phylogenetic diversity among patients with greater versus lower total arteriosclerotic CSVD burden, while Shannon and Simpson indices were similar [29]. Because this represented a within-CSVD severity comparison rather than a disease-versus-control contrast, it is presented separately in Table 3.

Beta diversity evidence was less directly comparable. The Sun Yat-sen shotgun study reported a Bray–Curtis community composition difference between arteriosclerotic CSVD and controls (adonis R^2^ = 0.08, *p* = 0.001) [26]. The Anhui glymphatic study also reported a Bray–Curtis difference across controls, WMHs without cognitive impairment, and WMHs with cognitive impairment (permutational multivariate analysis of variance *p* = 0.025) [24]. The Maoming study identified Jaccard-based community differences primarily between arteriosclerotic CSVD and arteriosclerotic CSVD with idiopathic normal pressure hydrocephalus [23]. Because dispersion testing was not reported, the PERMANOVA findings should be interpreted cautiously, as differences in dispersion may have contributed to the observed group separation. Other reports relied mainly on partial least-squares discriminant analysis or related visual discrimination approaches without conventional inferential beta diversity testing [28,29,32].

### 3.4. Taxonomic Composition and Differential Abundance

Taxonomic findings varied substantially among studies, and no single genus or species was consistently associated with CSVD across the available cohorts. The statistical support for individual findings also varied, ranging from multiplicity-corrected differential abundance results to nominal or unadjusted associations. For interpretation, taxonomic associations were considered across three broad levels of statistical support: (1) findings surviving multiplicity correction; (2) associations supported by adjusted multivariable analyses without matrix-wide multiplicity correction; and (3) exploratory, nominal, or unadjusted findings.

Proteobacteria- or Enterobacterales-related signals were reported in several more affected clinical or imaging phenotypes. The Sun Yat-sen shotgun cohort reported higher Proteobacteria together with several *Bacteroides*-related taxa in arteriosclerotic CSVD; species-level differential abundance testing in this study used Benjamini–Hochberg-adjusted p-values [26]. In the earlier Sun Yat-sen cohort, which had a non-overlapping documented recruitment period, Proteobacteria and Enterobacteriaceae-related taxa were associated with greater CSVD burden and WMHs in largely exploratory analyses without clear matrix-wide multiplicity correction [29]. The Guangdong cohort with cognitive impairment also showed descriptively higher Proteobacteria and Fusobacteria [32].

Reductions in selected obligate anaerobic or short-chain-fatty-acid-associated taxa were reported in several studies. Benjamini–Hochberg-adjusted species-level analyses identified lower *Faecalibacterium prausnitzii* and *Roseburia intestinalis* in the Sun Yat-sen shotgun cohort [26]. *Barnesiella*-related depletion was reported in Framingham in multiplicity-corrected analyses and in the severe-WMH Anhui cohort by LEfSe, with lower *Coprococcus* also reported in the latter [21,25].

Other taxonomic findings were less concordant. *Megasphaera* was reduced in the severe-WMH Anhui cohort but nominally increased in Wuxi, where the association did not survive false discovery rate correction. *Bacteroides*-related signals occurred in the GIMLET, Sun Yat-sen, and Anhui studies, but at different taxonomic levels, against different outcomes, and using different statistical approaches [22,24,26,27,28,30].

The Wuxi study reported false-discovery-rate-significant enrichment of *Parasutterella* and *Anaeroglobus*, whereas *Collinsella* and *Veillonella* appeared in unadjusted MRI–taxon correlations [28]. The Guangdong study reported lower *Prevotella-9* and higher *Fusobacterium* in LEfSe-based analyses without false discovery rate correction reported for these specific comparisons [32]. In Maoming, *Solobacterium* and *Thermus* were identified in false-discovery-rate-adjusted correlations with regional imaging measures [23]. Accordingly, multiplicity-corrected findings were given greater interpretive weight than isolated nominal or unadjusted taxonomic associations.

### 3.5. Associations with Structural Neuroimaging, Advanced Imaging, and Cognition

Direct microbiome–MRI associations were reported for WMHs, CMBs, lacunes, composite CSVD burden, and several advanced imaging phenotypes.

In Framingham, lower abundance of *Barnesiella intestinihominis* was associated with greater CSVD-related imaging burden. Higher abundance of *Pseudobutyrivibrio* and *Ruminococcus* was associated with lower WMH volume and lower PSMD, and both genera were also associated with better executive function [21]. Framingham analyses incorporated multivariable adjustment and Benjamini–Hochberg correction.

In GIMLET, the *Bacteroides*-dominant enterotype I was more frequent among participants with WMHs. In the later adjusted report, the association had an OR of 3.2 (95% CI 0.98–11.47; *p* = 0.06) after adjustment for age, sex, education, and vascular risk factors [22,30].

Several smaller studies reported associations between individual taxa and specific lesion types. In Wuxi, *Collinsella* correlated positively with total and periventricular Fazekas scores, whereas *Veillonella* correlated with lacune count [28]. These analyses were based on an unadjusted correlation matrix. In the earlier Sun Yat-sen cohort, Proteobacteria correlated positively with WMHs, and Enterobacteriaceae-related taxa were associated with greater total CSVD burden [29]. Multivariable adjustment was limited. In the severe-WMH Anhui report, differential microbial taxa did not correlate with Fazekas severity after false discovery rate correction [25].

The Anhui alpha diversity report provided the most detailed adjusted analysis of richness against individual structural outcomes. After adjustment for vascular risk factors, higher ln-transformed Chao1 was associated with fewer CMBs (exponentiated estimate 0.213, 95% confidence interval 0.093–0.487; *p* < 0.001) [31]. Higher Chao1 was also associated with lower modified composite CSVD burden. Associations with lacune count and WMH volume did not reach statistical significance. Representative direct microbiome–neuroimaging associations are summarized in Table 4, with effect estimates and 95% confidence intervals reported where available from the original studies.

Advanced neuroimaging studies extended the associations beyond conventional structural lesions. In Maoming, *Thermus* correlated with a higher deep medullary vein score (*r* = 0.271; false-discovery-rate-adjusted *p* = 0.031), whereas *Solobacterium* correlated inversely with deep and basal ganglia CMB counts [23]. These analyses did not include multivariable adjustment for vascular risk factors.

The larger Anhui cognition cohort examined neurovascular coupling measures. CBF–functional neurovascular coupling coefficients, fractional amplitude of low-frequency fluctuation, and degree centrality were lower in participants with CSVD and mild cognitive impairment than in those with CSVD without cognitive impairment [27]. After multiplicity correction, *Dialister* was inversely associated with selected neurovascular coupling indices [27]. The principal microbiome–neurovascular coupling models did not additionally account for WMH volume, which differed between the cognitive subgroups.

Community-level richness was also related to cognition in the potentially overlapping Anhui alpha diversity study. Higher Chao1 was associated with better Montreal Cognitive Assessment score, processing speed, and language after adjustment for age, sex, and education (β = 0.781, *p* = 0.028; β = 0.618, *p* = 0.036; and β = 0.529, *p* = 0.019, respectively) [31]. These associations were no longer statistically significant after additional adjustment for structural MRI markers or composite CSVD burden. This attenuation limits interpretation of these associations as independent microbiome–cognition relationships. Cross-sectional mediation analyses reported indirect effects through total burden for processing speed and language.

The Anhui glymphatic study examined microbiome relationships with several MRI-derived clearance measures [24]. White-matter-free water was higher and the index of diffusivity along the perivascular space (ALPS index) was lower in the cognitively impaired WMH group [24]. Cross-sectional mediation models linked *Acetivibrio* to Montreal Cognitive Assessment score through tetradecyldiethanolamine and the ALPS index, while an association in the opposite direction was reported for *Staphylococcus*. These analyses linked microbial, metabolic, imaging, and cognitive variables within the same multimodal framework. Because the mediation models were cross-sectional, the reported indirect effects should not be interpreted as evidence of temporal or causal mediation.

### 3.6. Mechanistic and Multi-Omics Evidence

Five reports provided complementary evidence involving microbial metabolism, immune activation, intestinal barrier function, microbiome–metabolite relationships, or experimental transfer [24,25,26,29,32]. Functional evidence was interpreted according to the underlying measurement modality. Shotgun metagenomics directly characterizes microbial gene content and pathway potential, and metabolomics directly measures metabolites, whereas functional profiles inferred from 16S rRNA data represent computational predictions based on taxonomic composition and were therefore treated as hypothesis-generating rather than as direct measurements of microbial function.

Metabolic and immune findings were prominent in the two independent Sun Yat-sen cohorts. Shotgun metagenomic and metabolomic profiling identified functional signals related to lipopolysaccharide biosynthesis and phenylalanine–tyrosine metabolism alongside the reported taxonomic differences in arteriosclerotic CSVD [26]. In the earlier study, circulating neutrophils from patients with arteriosclerotic CSVD showed increased interleukin-17A (IL-17A) expression. Fecal bacterial extracts from affected patients induced IL-17A production in healthy donor neutrophils through retinoic acid receptor-related orphan receptor gamma t (RORγt) signaling; pharmacological inhibition reversed this response. The extracts additionally increased transcription of proinflammatory, adhesion-related, and matrix metalloproteinase 8, 9, and 13 genes [29].

The Anhui studies provided complementary multi-omics and imaging evidence. In the severe-WMH cohort, *Haemophilus* and *Akkermansia* were linked to metabolites associated with aminoacyl-transfer RNA biosynthesis and to multimodal imaging and cognitive measures, although differential microbial and metabolite features were not associated with Fazekas severity after false discovery rate correction [25]. The later, potentially overlapping glymphatic study reported cross-sectional microbiota–metabolite–MRI–cognition mediation pathways involving the ALPS index [24].

The Guangdong study assessed intestinal permeability in patients with CSVD and cognitive impairment [32]. Among the three urinary permeability measures, only mannitol recovery was higher than in controls (29.15 ± 19.4% versus 11.85 ± 5.54%; *p* = 0.001); lactulose recovery and the lactulose/mannitol ratio did not differ. Higher mannitol recovery correlated with lower Mini-Mental State Examination scores, slower Trail Making Test Part B performance, and worse activities of daily living. MaAsLin analyses additionally linked higher mannitol recovery with lower *Parasutterella* and *Parabacteroides* and higher *Prevotella-7*.

In the experimental component of the same report, fecal microbiota transplantation from donors with CSVD and cognitive impairment increased gut permeability in mice, whereas locomotor and novel object recognition outcomes were unchanged [32]. Thus, the experiment reproduced an intestinal barrier phenotype but did not reproduce a detectable neurological or cognitive phenotype.

Collectively, these studies provide complementary evidence at several biological levels—including microbial functional pathways, immune activation, intestinal barrier measures, circulating metabolites, neurovascular measures, MRI-derived clearance phenotypes, ex vivo responses, and limited experimental transfer. These levels of evidence are biologically complementary but are not equivalent for causal inference.

## 4. Discussion

### 4.1. An Integrative Interpretation of the Current Evidence

The central finding of this review is that the microbiome–CSVD literature becomes more coherent when microbial associations are considered at different biological levels rather than as a search for a single disease-specific taxonomic signature. The available evidence shows qualitative convergence in the biological processes implicated across studies, although the degree of convergence has not been formally quantified. Richness-related measures show a recurrent community-level pattern, whereas taxonomic associations vary across MRI phenotypes. Complementary observational, multi-omics, ex vivo, and experimental studies implicate intestinal barrier, immune–metabolic, neurovascular, and perivascular clearance processes [21,22,23,24,25,26,27,28,29,30,31,32].

This pattern suggests that the relevant microbial phenotype in CSVD may be better conceptualized as an ecosystem state than as the presence or absence of a particular organism. Individual taxa may provide phenotype-specific signals, whereas community structure and collective microbial function may capture broader biological relationships with small vessel injury. Viewed in this way, the marked taxonomic heterogeneity of the literature does not necessarily contradict the existence of a meaningful microbiome–CSVD relationship.

This ecological framework also provides a useful basis for future research. Rather than asking whether a universal “CSVD microbiome” exists, the more informative questions may be which features of microbial ecosystem organization recur across independent cohorts, whether these features differ across CSVD phenotypes, and whether distinct microbial configurations converge on common biological effects. Current cross-sectional evidence cannot establish directionality, but it provides a set of testable hypotheses for longitudinal and mechanistic studies.

### 4.2. Microbial Richness as a Recurrent but Preliminary Ecological Signal

Among the ecological measures evaluated to date, microbial richness shows the most recurrent directional pattern across disease-versus-control cohort families. Chao/Chao1 was numerically lower in CSVD across four cohort families [26,28,31,32], with statistically significant differences in two. A potentially overlapping Anhui report provided additional within-family support [27]. Shannon and Simpson indices were less consistent. Taken together, these findings suggest a recurring richness-related signal, although the current evidence base is still too limited to regard it as an established CSVD-associated ecological phenotype.

Chao/Chao1 and related estimators primarily reflect richness, whereas Shannon and Simpson indices also incorporate the relative distribution of abundance among community members [33,35]. The available evidence therefore points more specifically toward altered richness than toward a generalized reduction in all dimensions of alpha diversity. Evenness itself remains insufficiently characterized because dedicated evenness measures have been reported inconsistently. Chao1 is particularly sensitive to the detection of rare taxa and therefore to sequencing depth and preprocessing choices. Differences in filtering, rarefaction, OTU-versus-ASV processing, and sequencing platform may consequently influence richness estimates and further limit direct comparison across studies [33,35].

This distinction moves the field beyond the broad and often imprecise concept of “microbial diversity”. A microbiome containing fewer detectable community members represents a different ecological state from one in which the same organisms are present but distributed differently in abundance. These ecological dimensions may also have different relationships with metabolic capacity, functional redundancy, resilience, and host–microbial interactions.

Microbial richness may therefore represent a candidate ecological feature associated with CSVD. Its biological interpretation is not necessarily linear, however. The report of greater richness in patients with higher rather than lower arteriosclerotic CSVD burden indicates that richness is unlikely to function as a simple severity scale and further argues against interpreting greater microbial richness as inherently beneficial [29]. Richness should therefore be considered a context-dependent ecological characteristic rather than a direct marker of microbiome health and may vary with disease stage, clinical phenotype, vascular risk, medication exposure, diet, or other host characteristics. Longitudinal studies with repeated microbiome and MRI measurements should determine how richness changes across the development and progression of small vessel disease and whether it provides information beyond established vascular risk factors.

Interpretation of these richness–imaging associations is also limited by neuroimaging heterogeneity across studies. Composite burden scores, continuous imaging measures, and binary imaging outcomes should not be regarded as interchangeable; recurrence of microbiome associations across these measures therefore reflects relationships with related CSVD phenotypes rather than replication of an identical neuroimaging endpoint.

### 4.3. Taxonomic Heterogeneity May Be Biologically Informative

No single genus or species has been consistently replicated across the available cohorts. Rather than being treated only as a negative finding, this heterogeneity may itself be informative.

CSVD is not a biologically uniform condition. WMHs, lacunes, CMBs, EPVSs, diffuse white matter injury, venous abnormalities, neurovascular dysfunction, and altered perivascular clearance represent related but distinct manifestations of cerebral small vessel pathology [6,7,36]. Microbial associations may therefore track individual components of this phenotype rather than the broad diagnostic label of CSVD. The observation that different microbial features associate with different MRI manifestations is consistent with such a phenotype-specific model.

This interpretation also fits the available richness data. In the most extensively adjusted analysis, higher Chao1 was associated with fewer CMBs and lower composite burden, whereas associations with lacune count and WMH volume were not statistically significant [31]. The relevant microbial signal may therefore differ not only between populations but also between components of cerebral small vessel injury within the same population.

Methodological variation provides a second explanation for taxonomic inconsistency. As summarized in Table 2, studies differ substantially in microbiome profiling, 16S target region, bioinformatic processing, taxonomic resolution, differential abundance methodology, multiple-testing correction, clinical sampling, and neuroimaging phenotyping. These factors can materially influence which taxa are identified. Variation in multiple-testing correction may contribute specifically to the apparent taxonomic heterogeneity because nominal or unadjusted associations are not directly comparable with findings that remained significant after multiplicity correction. Biological phenotype specificity and methodological heterogeneity are therefore likely to operate simultaneously.

Future studies should preserve individual imaging phenotypes rather than collapsing CSVD into a binary diagnosis whenever possible. Mapping microbial characteristics to defined hemorrhagic, ischemic, white matter, venous, neurovascular, and clearance-related phenotypes may ultimately be more informative than continuing to search for a universal CSVD-associated organism.

### 4.4. Qualitative Convergence of Candidate Biological Processes Across Evidence Levels

The available multi-omics, ex vivo, and experimental literature implicates several overlapping biological processes, although the strength of inference differs substantially across evidence levels. Human observational and multi-omics studies identify associations involving intestinal barrier alterations, microbial metabolic pathways, neurovascular abnormalities, and MRI-derived indices of perivascular clearance. Ex vivo experiments provide more direct evidence that patient-derived microbial material can influence IL-17A/RORγt-related immune responses. The limited animal evidence demonstrates transfer of selected biological effects, most notably altered intestinal permeability, but has not established a corresponding neurological or cognitive phenotype [24,25,26,29,32]. These findings identify biologically related processes relevant to small vessel integrity, but they do not establish a unified causal mechanism in human sporadic CSVD [37].

Taken together, these findings provide cross-level biological plausibility for a hypothesis-generating ecosystem-level framework. In this framework, taxonomically distinct microbial communities may be associated with overlapping microbial and host-response processes relevant to CSVD. Functional redundancy within the gut microbiome provides one plausible explanation for why taxonomic signatures may vary considerably while related metabolic, barrier, or immune processes recur across studies [38].

This framework also links the richness signal with the mechanistic evidence. Richness describes one aspect of community organization, whereas the biological consequences of that organization depend on the collective functional capacity of the ecosystem. Reduced richness may therefore mark a broader alteration in microbial community structure, functional redundancy, ecological resilience, or host–microbial signaling rather than acting as a pathogenic factor in isolation.

The implication is that convergence at the level of microbial function, microbial products, or host response may ultimately be more informative than recurrence of individual taxa. Studies integrating metagenomics with metabolomics, intestinal barrier assessment, immune profiling, vascular biomarkers, and quantitative neuroimaging could directly test whether taxonomically different communities are linked to shared biological pathways relevant to small vessel injury. Experimental studies should extend these observations using fecal microbiota transplantation from well-phenotyped donors and parallel assessment of intestinal and blood–brain barrier integrity, endothelial dysfunction, neuroinflammation, cerebral perfusion, white matter injury, and cognition. Rescue experiments using candidate microbes or metabolites could further test the specificity and reversibility of implicated pathways.

**Figure 1:**
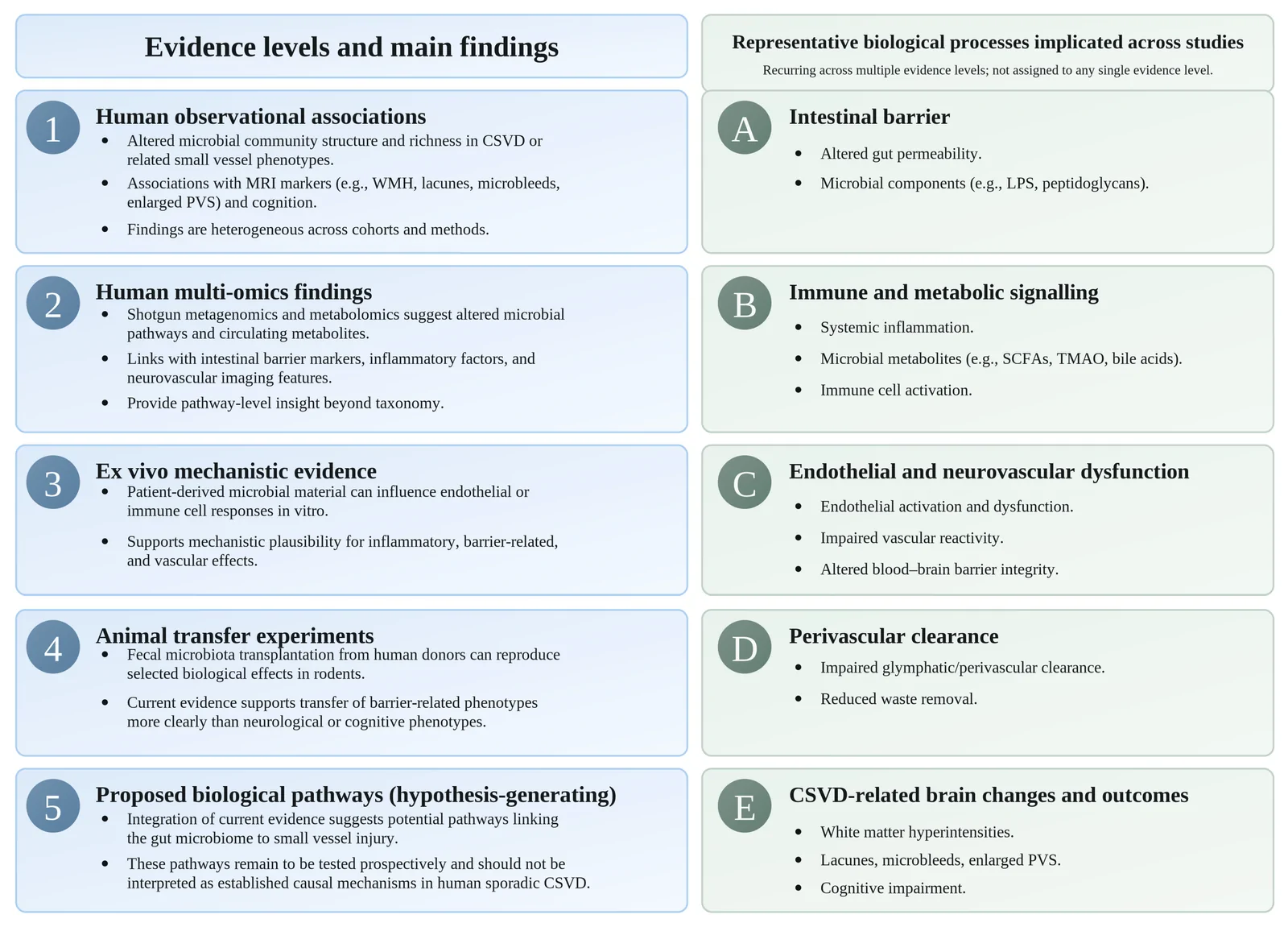
Levels of evidence and representative biological processes linking the gut microbiome with cerebral small vessel disease (CSVD). The left panel distinguishes human observational associations, human multi-omics findings, ex vivo mechanistic evidence, animal transfer experiments, and proposed biological pathways. The right panel summarizes representative biological processes that recur across multiple evidence levels and are not assigned to any single evidence category. Evidence levels are complementary but not equivalent for causal inference; proposed pathways remain hypothesis-generating rather than established causal mechanisms in human sporadic CSVD.

The complementary evidence levels and representative biological processes implicated across studies are summarized in Figure 1.

This ecosystem-level framework remains to be tested prospectively. Its value at present is to organize recurring biological associations across complementary evidence levels and to define specific pathways that can be evaluated in longitudinal and experimental studies.

### 4.5. Nutritional Relevance and the Modifiability of the Microbial Ecosystem

Diet is an important modifiable determinant of the gut microbial ecosystem. Human intervention studies have shown that dietary exposures can alter microbial community composition and function, and selected interventions may also influence microbial diversity and systemic immune profiles [15,39]. This is particularly relevant to the present findings because several of the recurring signals in CSVD involve community-level ecological features, including microbial richness, rather than a single reproducible taxonomic signature. However, the observational associations identified in CSVD do not establish that altered microbial richness or other ecosystem features contribute causally to small vessel injury, nor do they demonstrate that modifying these features through diet will alter disease progression. This distinction provides a clear direction for prospective research. Future studies could test whether targeted dietary interventions modify microbial richness or other reproducible ecological and functional characteristics and, importantly, whether such changes are accompanied by alterations in intestinal barrier function, immune–metabolic signaling, vascular physiology, quantitative MRI markers, or cognition [40,41,42].

### 4.6. Strengths and Limitations

This review provides a structured interpretation of a fragmented literature by separating several levels of microbiome–CSVD evidence that have often been considered together. It distinguishes richness-focused measures from broader alpha diversity indices, preserves individual MRI phenotypes and taxonomic rank, differentiates inferential community-level analyses from descriptive discrimination methods, and considers human observational and multi-omics, ex vivo, and experimental findings as distinct but complementary levels of evidence. Consideration of the underlying participant cohort rather than publication count also reduces the risk of interpreting overlapping reports as independent replication.

The principal limitations arise from the available evidence base. Human microbiome–CSVD studies remain predominantly cross-sectional, and many cohorts are small, clinically selected, and geographically concentrated, with most reports originating from Chinese hospital-based populations. Consequently, large population-based and geographically diverse replication remains limited, which constrains generalizability to broader populations. Adjustment for major microbiome-related and vascular confounders—including diet and geographic dietary patterns, BMI/adiposity, diabetes, hypertension, medication and antibiotic exposure, smoking, alcohol consumption, physical activity, and constipation—was inconsistent across studies. Residual confounding by these factors may therefore contribute to between-cohort differences in both ecological and taxonomic associations.

At the review level, no separate systematic search of grey literature, preprint servers, or backward citations was performed, and relevant unpublished or non-indexed reports may therefore have been missed. Publication bias cannot be excluded, and despite structured eligibility criteria and review of study selection by two authors, some selection bias may remain. No quantitative synthesis was undertaken because of the small number of cohort families, potential participant overlap, and substantial methodological and phenotypic heterogeneity.

Methodological diversity across microbiome profiling, bioinformatic processing, statistical analysis, and neuroimaging also limits direct cross-study comparison. This heterogeneity is particularly relevant to taxonomic findings and makes it difficult to distinguish study-specific analytical effects from genuine biological differences. Study-level methodological limitations were considered in relation to study design, sample selection, confounder adjustment, multiple-testing procedures, microbiome methodology, neuroimaging definitions, and potential cohort overlap.

These limitations primarily constrain causal interpretation rather than the identification of recurring patterns. The present evidence suggests a recurrent richness-related signal, alongside phenotype-specific rather than universal taxonomic associations and an ecosystem-level model of convergent microbial–host biology. The limited number of independent cohorts and variable statistical support indicate that the richness-related pattern requires further replication. Establishing whether these features precede, accompany, or result from cerebral small vessel injury will require longitudinal, multicenter studies with repeated stool sampling and serial quantitative MRI. Future studies should also integrate shotgun metagenomics, metabolomics, intestinal barrier and inflammatory biomarkers, and standardized assessment of diet, medication and antibiotic exposure, and other major microbiome determinants.

## 5. Conclusions

Current evidence does not support a single taxonomic signature of sporadic CSVD. Richness-based measures, particularly Chao/Chao1, show a recurrent numerical direction across several disease-versus-control cohorts, but statistical support is inconsistent, and the finding should be regarded as preliminary. Taxon-specific associations are more heterogeneous and appear to vary across MRI-defined phenotypes.

Complementary multi-omics, ex vivo, and experimental findings implicate intestinal barrier, immune–metabolic, neurovascular, and perivascular clearance processes but do not establish causality in human sporadic CSVD. These observations support a hypothesis-generating ecological framework in which microbial function and host response may ultimately prove more informative than individual taxa. Because diet is a modifiable determinant of the gut microbiome, nutritional strategies represent a plausible direction for future research; however, no CSVD-specific dietary or microbiome-directed intervention has yet been shown to modify disease progression or MRI-defined outcomes. Whether any microbial or functional features are reproducible across independent cohorts, temporally related to CSVD progression, and responsive to nutritional or microbiome-directed intervention remains to be determined in longitudinal and interventional studies.

## Figures and Tables

**Table 1:** Characteristics of the available reports and cohort relationships.

Study/Setting	Design and Population	*N*	Microbiome Assessment	MRI/CSVD Phenotype	Cohort Relationship
Fongang et al., 2023 USA; Framingham [21]	Population-based cross-sectional secondary analysis; adults with stool, MRI and cognition	972	16S V4; MiSeq; QIIME2; 97% OTU	WMH volume; PSMD	Distinct Framingham cohort
Saji et al., 2019 Japan; GIMLET/NCGG [22]	Memory clinic cross-sectional study; MCI 61, normal cognition 21	82	16S-based T-RFLP; enterotypes; F/B ratio	WMH; silent lacunar infarct; CMB	Overlaps Saji et al., 2021 [30] (same GIMLET framework)
Cai et al., 2026 China; Maoming [23]	Three-group study; aCSVD 45, aCSVD-iNPH 20, HCs 30	95	16S V3–V4; MiSeq/NovaSeq; LEfSe	Fazekas/WMH; lacunes; CMB; DMV	Distinct Maoming cohort
Xu et al., 2026 China; Anhui [24]	HC 56, WMHs without cognitive impairment 40, WMHs with cognitive impairment 49	145	16S V4; NovaSeq; ASV/UNOISE3; multi-omics	WMH volume/Fazekas; glymphatic MRI proxies	Probable Anhui overlap
Song et al., 2024 China; Anhui [25]	Severe WMH-predominant CSVD 37 and matched HCs 46	83	16S V4; NovaSeq; ASV; metabolomics	Severe WMH; multimodal MRI	Probable Anhui overlap
Huang et al., 2024 China; Sun Yat-sen [26]	aCSVD 37 and HCs 30; shotgun multi-omics case–control study	67	Shotgun metagenomics; NovaSeq; pathway profiling	MRI-defined aCSVD; total burden	Separate from Cai et al., 2021 [29]; non-overlapping recruitment
Zhang et al., 2024 China; Anhui [27]	HCs 40, CSVD-NCI 61, CSVD-MCI 43	144	16S V4; NovaSeq; 97% OTU	WMH volume/Fazekas; lacunes; CMB; neurovascular coupling	Potential overlap with Huang et al., 2024 [31] (alpha-diversity report)
Shi et al., 2023 China; Wuxi [28]	CSVD 64 and community HCs 18	82	16S V3–V4; QIIME2; OTU-based reporting	Fazekas; lacunes; CMB; EPVS; ALFF	Distinct Wuxi cohort
Cai et al., 2021 China; Sun Yat-sen [29]	aCSVD 55 and HCs 62; aCSVD burden 0–1 vs. 2–4	117	16S V3–V4; MiSeq; 99% OTU	Standard 0–4 CSVD burden: WMH, lacunes, CMB, PVS	Separate from Huang et al., 2024 [26]; non-overlapping recruitment
Saji et al., 2021 Japan; GIMLET/NCGG [30]	Memory clinic cross-sectional analysis; MCI 64, non-MCI 23	87	T-RFLP; enterotypes; F/B ratio; fecal metabolites	1.5 T MRI; WMH, SLI, CMB, EPVS; total SVD score 0–4	Overlaps Saji et al., 2019 [22]
Huang et al., 2024 China; Anhui [31]	HCs 40, CSVD-NCI 43, CSVD-MCI 35	118	16S V4; NovaSeq; 97% OTU	WMH volume/Fazekas; lacunes; CMB; modified 0–8 burden	Potential overlap with Zhang et al., 2024 [27]
Chen et al., 2026 China; Guangdong [32]	CSVD with cognitive impairment 21 and HCs 20	41	Human stool 16S; exact human amplicon/platform incompletely reported	Periventricular + deep WMH; Fazekas sum 0–6	Distinct Guangdong cohort; mouse FMT analyzed separately

Abbreviations: aCSVD, arteriosclerotic cerebral small vessel disease; ALFF, amplitude of low-frequency fluctuation; ASV, amplicon sequence variant; CMB, cerebral microbleed; CSVD, cerebral small vessel disease; CSVD-MCI, cerebral small vessel disease with mild cognitive impairment; CSVD-NCI, cerebral small vessel disease without cognitive impairment; DMV, deep medullary vein; EPVS, enlarged perivascular space; F/B, Firmicutes/Bacteroidetes ratio; FMT, fecal microbiota transplantation; GIMLET, Gerontological Investigation of Microbiome: a Longitudinal Estimation Study; HCs, healthy controls; iNPH, idiopathic normal pressure hydrocephalus; LEfSe, linear discriminant analysis effect size; MCI, mild cognitive impairment; MRI, magnetic resonance imaging; NCGG, National Center for Geriatrics and Gerontology; NCI, no cognitive impairment; OTU, operational taxonomic unit; PSMD, peak width of skeletonized mean diffusivity; PVS, perivascular space; QIIME, Quantitative Insights Into Microbial Ecology; SLI, silent lacunar infarct; SVD, small vessel disease; T-RFLP, terminal restriction fragment length polymorphism; WMH, white matter hyperintensity. Because several publications arose from overlapping cohorts, *N* values must not be summed across reports.

**Table 2:** Major sources of methodological heterogeneity in microbiome–CSVD research.

Domain	Variation Across Studies	Consequence for Interpretation
Microbiome profiling	T-RFLP, 16S rRNA sequencing, shotgun metagenomics	Different taxonomic and functional resolution limits direct comparison of microbial findings.
16S sequencing strategy	V3–V4 vs. V4 regions; MiSeq vs. NovaSeq	Amplicon region and sequencing platform may influence detected community composition.
Bioinformatic processing	OTU vs. ASV approaches; clustering thresholds; different pipelines and taxonomic databases	The same biological community may yield different taxonomic assignments and abundance estimates.
Alpha diversity metrics	Chao/Chao1, ACE, observed richness, Shannon, Simpson, Pielou evenness, Faith PD	These indices quantify different ecological properties; “alpha diversity” is therefore not a uniform construct.
Beta diversity analysis	Bray–Curtis, UniFrac, Jaccard; PERMANOVA vs. PLS-DA or descriptive ordination	Visual group separation and inferential evidence of community-level differences are not equivalent.
Differential abundance statistics	Unadjusted comparisons, correlations, regression, LEfSe, and other approaches	Different analytical strategies can generate different sets of candidate taxa.
Multiple-testing control	FDR/Bonferroni correction vs. nominal *p*-values	Nominal taxonomic associations may have substantially lower reproducibility.
Confounder adjustment	From unadjusted analyses to multivariable vascular risk models	Differences in age, vascular risk, adiposity, medication, and lifestyle adjustment complicate comparison.
Clinical populations	Population-based cohorts, hospital controls, memory clinic cohorts, CSVD with/without cognitive impairment	Study populations represent different clinical and disease spectra.
CSVD definition	Binary CSVD diagnosis, WMH-predominant disease, burden strata, continuous imaging measures	Studies may not be examining the same underlying CSVD phenotype.
WMH assessment	Fazekas grading, volumetry, phenotype-based inclusion	Ordinal and quantitative WMH measures are not directly interchangeable.
Individual MRI phenotypes	WMHs, lacunes, CMBs, EPVSs, deep medullary veins, and advanced markers	Different endpoints represent distinct components of small vessel injury.
Composite CSVD scores	Conventional 0–4 score vs. modified 0–8 approaches	Different weighting and lesion composition limit direct comparison of total burden associations.
Advanced neuroimaging	PSMD, neurovascular coupling, resting-state measures, diffusion/glymphatic proxies	These measures interrogate different structural and functional biological processes.
Timing and environmental exposure	Variable or incompletely reported stool–MRI interval, diet, antibiotics, and medications	Temporal variability and external microbiome determinants may contribute to between-study differences.
Cohort overlap	Multiple publications from shared or potentially shared participant samples	Apparent replication across publications may not represent independent evidence.

Abbreviations: ACE, abundance-based coverage estimator; ASV, amplicon sequence variant; CMB, cerebral microbleed; CSVD, cerebral small vessel disease; EPVSs, enlarged perivascular spaces; FDR, false discovery rate; LEfSe, linear discriminant analysis effect size; MRI, magnetic resonance imaging; OTU, operational taxonomic unit; PD, phylogenetic diversity; PERMANOVA, permutational multivariate analysis of variance; PLS-DA, partial least-squares discriminant analysis; PSMD, peak width of skeletonized mean diffusivity; rRNA, ribosomal RNA; T-RFLP, terminal restriction fragment length polymorphism; WMH, white matter hyperintensity.

**Table 3:** Community-level alpha diversity findings across the principal cohort families.

Cohort/Report	Clinical Contrast	Richness Finding	Shannon Finding	Cohort Independence/Statistical Context
Disease or group comparison studies				
Maoming/Cai 2026 [23]	aCSVD, aCSVD-iNPH and HC	No statistically significant group difference reported	No significant group difference reported	Distinct Maoming cohort; FDR-corrected group comparisons/correlations; primarily unadjusted analyses.
Sun Yat-sen/Huang 2024 multi-omics [26]	aCSVD vs. HC	Chao1 lower in aCSVD; *p* = 0.0027	Lower in aCSVD; *p* = 0.0053	Distinct Sun Yat-sen cohort; case–control comparison; limited adjusted modeling.
Anhui/Huang 2024 alpha [31]	CSVD vs. HC	Chao1 lower; *p* < 0.001; exact raw and ln-transformed values reported	No difference; *p* = 0.493 for ln-transformed values	Preferred alpha diversity report within the potentially overlapping Anhui family; adjusted MRI association models reported separately.
Wuxi/Shi 2023 [28]	CSVD vs. HC	Chao1 lower trend; *p* = 0.077	Lower in CSVD; *p* = 0.033	Distinct Wuxi cohort; largely unadjusted analyses; multiple-testing details limited.
Guangdong/Chen 2026 [32]	CSVD-CI vs. HC	Chao 247.02 ± 90.21 vs. 295.82 ± 109.13; *p* = 0.32	2.88 ± 0.62 vs. 3.07 ± 0.71; *p* = 0.24	Distinct but small Guangdong cohort; unadjusted case–control comparison.
Population-based continuous MRI association				
Framingham/Fongang 2023 [21]	Population-based continuous MRI burden	No significant global alpha diversity association	No consistent association	Distinct Framingham cohort; multivariable continuous outcome analysis; not a case–control contrast.
Within-CSVD severity analysis				
Sun Yat-sen/Cai 2021 [29]	Higher vs. lower total burden within aCSVD	Chao1, observed OTU and Faith PD higher with greater burden	No clear difference	Distinct Sun Yat-sen cohort; within-disease severity comparison; mostly unadjusted discovery analyses.

Abbreviations: aCSVD, arteriosclerotic cerebral small vessel disease; aCSVD-iNPH, arteriosclerotic cerebral small vessel disease with idiopathic normal pressure hydrocephalus; CSVD, cerebral small vessel disease; CSVD-CI, cerebral small vessel disease with cognitive impairment; FDR, false discovery rate; HC, healthy control; iNPH, idiopathic normal pressure hydrocephalus; MRI, magnetic resonance imaging; OTU, operational taxonomic unit; PD, phylogenetic diversity.

**Table 4:** Representative direct microbiome–neuroimaging associations.

Cohort/Report	Microbiome Exposure	MRI Outcome	Principal Result	Adjustment/Multiplicity/Cohort Context
Framingham/Fongang 2023 [21]	Taxon abundance	WMH volume; PSMD	Lower *Barnesiella intestinihominis* abundance was associated with greater imaging burden; higher *Pseudobutyrivibrio* and *Ruminococcus* abundance was associated with lower WMHs/PSMD.	Multivariable analysis; BH correction; distinct population-based Framingham cohort.
GIMLET/Saji 2021 [30]	*Bacteroides*-dominant enterotype I	WMH	Adjusted OR 3.2 (95% CI 0.98–11.47), *p* = 0.06.	Adjusted for age, sex, education and vascular risk factors; 23 WMH events; overlaps Saji et al., 2019 [22].
Maoming/Cai 2026 [23]	*Solobacterium*; *Thermus*	Deep/basal ganglia CMB; DMV score	*Solobacterium* inversely correlated with deep (*r* = −0.292; FDR *p* = 0.018) and basal ganglia CMB (*r* = −0.292; FDR *p* = 0.019); *Thermus* positively correlated with DMV score (*r* = 0.271; FDR *p* = 0.031).	FDR-adjusted correlations; no multivariable vascular risk adjustment; distinct Maoming cohort.
Wuxi/Shi 2023 [28]	*Collinsella*; *Veillonella*	Fazekas; lacune count	*Collinsella* positively associated with total/periventricular Fazekas; *Veillonella* positively associated with lacune count.	Exploratory unadjusted correlations; multiple-testing details limited; distinct Wuxi cohort.
Sun Yat-sen/Cai 2021 [29]	Proteobacteria; Enterobacteriaceae lineage	WMH; total CSVD burden	Proteobacteria correlated positively with WMH; Enterobacteriaceae-related taxa abundance was associated with greater CSVD burden.	Exploratory, largely unadjusted analysis; distinct from Huang et al., 2024 [26], based on non-overlapping recruitment.
Anhui/Huang 2024 alpha [31]	ln(Chao1)	CMB count; modified burden; lacunes; WMH volume	Full model: CMB estimate 0.213 (0.093–0.487), *p* < 0.001; burden β = −0.393 (−0.630 to −0.155), *p* = 0.002; lacunes and WMHV nonsignificant.	Adjusted for age, sex and vascular risk factors; potential Anhui overlap; modified 0–8 burden.

Abbreviations: BH, Benjamini–Hochberg; CI, confidence interval; CMB, cerebral microbleed; CSVD, cerebral small vessel disease; DMV, deep medullary vein; FDR, false discovery rate; GIMLET, Gerontological Investigation of Microbiome: a Longitudinal Estimation Study; MRI, magnetic resonance imaging; OR, odds ratio; PSMD, peak width of skeletonized mean diffusivity; WMH, white matter hyperintensity; WMHV, white matter hyperintensity volume.

## Data Availability

No new datasets were generated for this review. All information analyzed is derived from the cited original publications and their Supplementary Materials.

## Supplementary Materials

The following supporting information can be downloaded at https://www.mdpi.com/article/10.3390/nu18203421/s1, Figure S1: Study selection flow diagram; Table S1: Complete database search strategies and search counts; Table S2: Report-level full-text eligibility assessment, exclusions, and related reports; Table S3: Cohort overlap classification matrix; Table S4: Detailed study characteristics, microbiome methods, and MRI phenotypes; Table S5: Alpha- and beta diversity findings; Table S6: Taxon–MRI/CSVD association and taxonomic hierarchy map; Table S7: JBI appraisal of included analytical cross-sectional reports.

## References

[1] Ungvari A., Kállai A., Stankovics L., Lendvai-Emmert D., Gulej R., Pal E., Patai R., Csik B., Fekete M., Lipecz Á. (2025). Age-related alterations of cerebral autoregulation. Life.

[2] Kallai A., Ungvari A., Csaban D., Orfi Z., Lehoczki A., Harasztdombi J., Yabluchanskiy A., Benyó Z., Szappanos Á., Tarantini S. (2025). Clonal hematopoiesis of indeterminate potential (CHIP) in cerebromicrovascular aging: Implications for vascular contributions to cognitive impairment and dementia (VCID). GeroScience.

[3] World Health Organization Ageing and Health. https://www.who.int/news-room/fact-sheets/detail/ageing-and-health.

[4] GBD 2021 Nervous System Disorders Collaborators (2024). Global, regional, and national burden of disorders affecting the nervous system, 1990-2021: A systematic analysis for the Global Burden of Disease Study 2021. Lancet Neurol..

[5] World Health Organization Dementia. https://www.who.int/news-room/fact-sheets/detail/dementia.

[6] Rost N.S., Etherton M. (2020). Cerebral Small Vessel Disease. Continuum.

[7] Wardlaw J.M., Smith C., Dichgans M. (2019). Small vessel disease: Mechanisms and clinical implications. Lancet Neurol..

[8] De Silva T.M., Faraci F.M. (2020). Contributions of Aging to Cerebral Small Vessel Disease. Annu. Rev. Physiol..

[9] Debette S., Markus H.S. (2010). The clinical importance of white matter hyperintensities on brain magnetic resonance imaging: Systematic review and meta-analysis. BMJ.

[10] Liu J., Daugherty A.M., Dahle C.L., Ghisletta P., Raz N. (2026). Seven-year progression of white matter hyperintensities and age-related cognitive change in healthy middle-aged and older adults. GeroScience.

[11] Fekete M., Varga P., Ungvari Z., Fekete J.T., Buda A., Szappanos Á., Lehoczki A., Mózes N., Grosso G., Godos J. (2025). The role of the Mediterranean diet in reducing the risk of cognitive impairement, dementia, and Alzheimer’s disease: A meta-analysis. Geroscience.

[12] Kapasi A., Schneider J.A. (2016). Vascular contributions to cognitive impairment, clinical Alzheimer’s disease, and dementia in older persons. Biochim. Biophys. Acta.

[13] Verheggen I.C., de Jong J.J., van Boxtel M.P., Gronenschild E.H., Palm W.M., Postma A.A., Jansen J.F., Verhey F.R., Backes W.H. (2020). Increase in blood–brain barrier leakage in healthy, older adults. Geroscience.

[14] Gulej R., Patai R., Ungvari A., Kallai A., Tarantini S., Yabluchanskiy A., Huffman D.M., Conboy M.J., Conboy I.M., Kivimäki M. (2025). Impacts of systemic milieu on cerebrovascular and brain aging: Insights from heterochronic parabiosis, blood exchange, and plasma transfer experiments. Geroscience.

[15] Wastyk H.C., Fragiadakis G.K., Perelman D., Dahan D., Merrill B.D., Yu F.B., Topf M., Gonzalez C.G., Van Treuren W., Han S. (2021). Gut-microbiota-targeted diets modulate human immune status. Cell.

[16] Zou X., Wang L., Xiao L., Wang S., Zhang L. (2022). Gut microbes in cerebrovascular diseases: Gut flora imbalance, potential impact mechanisms and promising treatment strategies. Front. Immunol..

[17] Che Mohd Nassir C.M.N., Che Ramli M.D., Mohamad Ghazali M., Jaffer U., Abdul Hamid H., Mehat M.Z., Hein Z.M. (2024). The Microbiota-Gut-Brain Axis: Key Mechanisms Driving Glymphopathy and Cerebral Small Vessel Disease. Life.

[18] Fekete M., Lehoczki A., Major D., Fazekas-Pongor V., Csípő T., Tarantini S., Csizmadia Z., Varga J.T. (2024). Exploring the influence of gut–brain axis modulation on cognitive health: A comprehensive review of prebiotics, probiotics, and symbiotics. Nutrients.

[19] Kryczyk-Poprawa A., Rząsa-Duran E., Varga J.T., Lehoczki A., Zábó V., Fazekas-Pongor V., Major D., Csípő T., Szappanos Á., Lipécz Á. (2026). Gut Microbiota-Targeted Nutrition for Healthy Aging: Mechanistic Roles of Polyphenols and Dietary Fiber in Geroscience. Nutrients.

[20] Xie J., Zhang J., Zhang L., Chen X. (2026). Exercise prescription for mood and cognition: Targeting the microbiota-gut-brain axis through short-chain fatty acids. Front. Microbiol..

[21] Fongang B., Satizabal C., Kautz T.F., Wadop Y.N., Muhammad J.A.S., Vasquez E., Mathews J., Gireud-Goss M., Saklad A.R., Himali J. (2023). Cerebral small vessel disease burden is associated with decreased abundance of gut Barnesiella intestinihominis bacterium in the Framingham Heart Study. Sci. Rep..

[22] Saji N., Murotani K., Hisada T., Tsuduki T., Sugimoto T., Kimura A., Niida S., Toba K., Sakurai T. (2019). The relationship between the gut microbiome and mild cognitive impairment in patients without dementia: A cross-sectional study conducted in Japan. Sci. Rep..

[23] Cai J., Feng K., Chen J., Liang L., Wu Q., He Q., Zhang X., Lei J., Mai J., Li Y. (2026). Association between gut microbiota and imaging biomarkers in arteriosclerotic cerebral small vessel disease with idiopathic normal pressure hydrocephalus. Front. Neurosci..

[24] Xu X., Zhou X., Zhang M., Fang J., Yan Z., Zhu X., Yu Y., Zhu J. (2026). Resolving cognitive heterogeneity in white matter hyperintensities through integrated analyses of microbiome, metabolome, and brain glymphatic function. Alzheimers Dement..

[25] Song Y., Zhou X., Zhao H., Zhao W., Sun Z., Zhu J., Yu Y. (2024). Characterizing the role of the microbiota-gut-brain axis in cerebral small vessel disease: An integrative multi-omics study. Neuroimage.

[26] Huang J., Liu S., Li P., Wei L., Lin G., Lin J., Luo Y., Liu Y., Mao Y., Ruan H. (2024). Multi-omics analysis of gut-brain axis reveals novel microbial and neurotransmitter signatures in patients with arteriosclerotic cerebral small vessel disease. Pharmacol. Res..

[27] Zhang W., Yu X., Wei M., Zhou J., Zhou Y., Zhou X., Zhao K., Zhu X. (2024). The influence of alterations in the composition of intestinal microbiota on neurovascular coupling and cognitive dysfunction in individuals afflicted with CSVD. Brain Res. Bull..

[28] Shi Y., Zhao E., Li L., Zhao S., Mao H., Deng J., Ji W., Li Y., Gao Q., Zeng S. (2023). Alteration and clinical potential in gut microbiota in patients with cerebral small vessel disease. Front. Cell. Infect. Microbiol..

[29] Cai W., Chen X., Men X., Ruan H., Hu M., Liu S., Lu T., Liao J., Zhang B., Lu D. (2021). Gut microbiota from patients with arteriosclerotic CSVD induces higher IL-17A production in neutrophils via activating RORgammat. Sci. Adv..

[30] Saji N., Murotani K., Hisada T., Tsuduki T., Sugimoto T., Kimura A., Niida S., Toba K., Sakurai T. (2021). The Association between Cerebral Small Vessel Disease and the Gut Microbiome: A Cross-Sectional Analysis. J. Stroke Cerebrovasc. Dis..

[31] Huang C., Zhang W., Shen Z., Li M., Yin J., Tang Y., Zhou X., Zhu X., Sun Z. (2024). The association between alpha diversity of gut microbiota, neuroimaging markers and cognitive function in cerebral small vessel disease. Brain Res..

[32] Chen Y., Jie W., Xu Y., Chen X., Zhu S., Ma Y., Hu L., Chen C., Liu B., Xu D. (2026). Correlation study between gut microbiota and intestinal permeability in cerebral small vessel disease. J. Alzheimers Dis..

[33] Knight R., Vrbanac A., Taylor B.C., Aksenov A., Callewaert C., Debelius J., Gonzalez A., Kosciolek T., McCall L.I., McDonald D. (2018). Best practices for analysing microbiomes. Nat. Rev. Microbiol..

[34] Nearing J.T., Douglas G.M., Hayes M.G., MacDonald J., Desai D.K., Allward N., Jones C.M.A., Wright R.J., Dhanani A.S., Comeau A.M. (2022). Microbiome differential abundance methods produce different results across 38 datasets. Nat. Commun..

[35] Willis A.D. (2019). Rarefaction, Alpha Diversity, and Statistics. Front. Microbiol..

[36] van Dinther M., Voorter P.H.M., Zhang E., van Kuijk S.M.J., Jansen J.F.A., van Oostenbrugge R.J., Backes W.H., Staals J. (2024). The neurovascular unit and its correlation with cognitive performance in patients with cerebral small vessel disease: A canonical correlation analysis approach. Geroscience.

[37] Fekete M., Liotta E.M., Molnar T., Fülöp G.A., Lehoczki A. (2025). The role of atrial fibrillation in vascular cognitive impairment and dementia: Epidemiology, pathophysiology, and preventive strategies. Geroscience.

[38] Louca S., Polz M.F., Mazel F., Albright M.B.N., Huber J.A., O’Connor M.I., Ackermann M., Hahn A.S., Srivastava D.S., Crowe S.A. (2018). Function and functional redundancy in microbial systems. Nat. Ecol. Evol..

[39] David L.A., Maurice C.F., Carmody R.N., Gootenberg D.B., Button J.E., Wolfe B.E., Ling A.V., Devlin A.S., Varma Y., Fischbach M.A. (2014). Diet rapidly and reproducibly alters the human gut microbiome. Nature.

[40] Chaudhari D.S., Jain S., Yata V.K., Mishra S.P., Kumar A., Fraser A., Kociolek J., Dangiolo M., Smith A., Golden A. (2023). Unique trans-kingdom microbiome structural and functional signatures predict cognitive decline in older adults. Geroscience.

[41] Zachariou V., Pappas C., Bauer C.E., Shao X., Liu P., Lu H., Wang D.J., Gold B.T. (2024). Regional differences in the link between water exchange rate across the blood–brain barrier and cognitive performance in normal aging. Geroscience.

[42] Escalante J., Artaiz O., Diwakarla S., McQuade R.M. (2025). Leaky gut in systemic inflammation: Exploring the link between gastrointestinal disorders and age-related diseases. GeroScience.

