# Supplementary material for: Gut Microbiome in Sporadic Cerebral Small Vessel Disease: MRI-Defined Phenotypes, Mechanistic Evidence, and Nutritional Implications for Brain Aging
Source: Nutrients. 2026 Oct 18;18(20):3421. doi: 10.3390/nu18203421 (PMC13708570; doi:10.3390/nu18203421)
Supplement: Supplementary file 1 [file nutrients-18-03421-s001.zip › nutrients-4588742-supplementary.pdf]

SUPPLEMENTARY MATERIALS

Gut Microbiome in Sporadic Cerebral Small Vessel Disease: MRI-Defined Phenotypes, Mechanistic Evidence, and Nutritional Implications for Brain Aging

Contents

- Figure S1. Study-selection flow diagram
- Table S1. Complete database search strategies and search counts
- Table S2. Report-level full-text eligibility assessment, exclusions, and related reports
- Table S3. Cohort-overlap classification matrix
- Table S4. Detailed study characteristics, microbiome methods, and MRI phenotypes
- Table S5. Alpha- and beta-diversity findings
- Table S6. Taxon–MRI/CSVD association and taxonomic hierarchy map
- Table S7. JBI appraisal of included analytical cross-sectional reports
- Supplementary abbreviations

Reference numbers in Supplementary Table S3 correspond to the reference list of the main manuscript. The supplementary tables otherwise identify primary reports by author, year, DOI, or source URL; no separate supplementary reference list is required.

Figure S1. Study-selection flow diagram

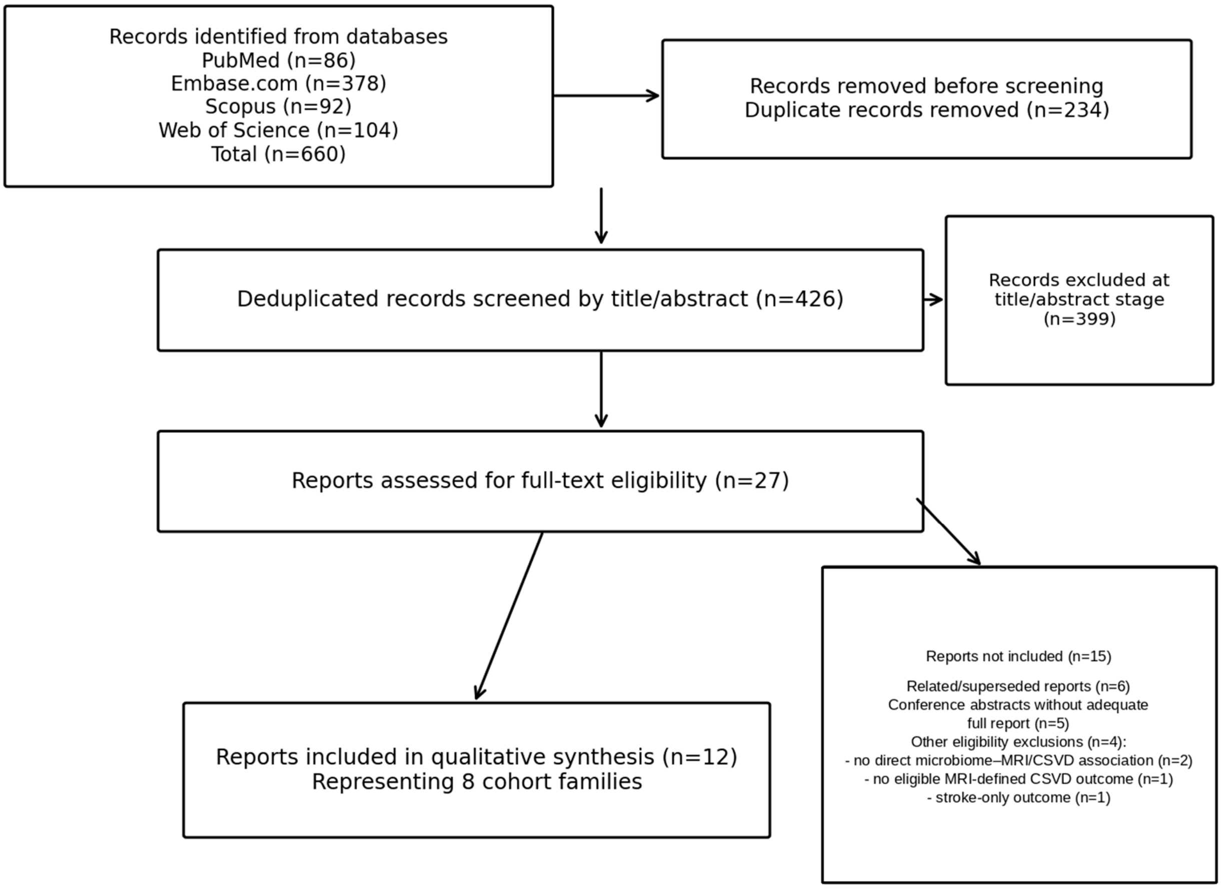

The report was the unit of study selection; the underlying participant cohort or cohort family was the unit of evidence for synthesis. Five abstract-only conference reports were not retained because no corresponding full-text report was identified and the available information was insufficient for reliable extraction and appraisal.

Table S1. Complete database search strategies and search counts

All searches were executed on 4 August 2026 from database inception without date or language restrictions and without automated human, adult, animal, or study-design filters. The Embase search was run via Embase.com.

Database	Platform/interface	Coverage	Search date	Records	Limits/notes
MEDLINE	PubMed	Inception to 4 Aug 2026	4 Aug 2026	86	No date, language, human, adult, animal, or study-design filters
Embase	Embase.com	Inception to 4 Aug 2026	4 Aug 2026	378	No date, language, human, adult, animal, conference, or publication-type filters
Scopus	Scopus	Inception to 4 Aug 2026	4 Aug 2026	92	TITLE-ABS-KEY; no date, language, or document-type limits
Web of Science	Core Collection	All available years to 4 Aug 2026	4 Aug 2026	104	Topic search; no date, language, or document-type limits

Table S1 — PubMed exact executed strategy

```
(
  "Gastrointestinal Microbiome"[Mesh]
  OR "Brain-Gut Axis"[Mesh]
  OR "gut microbiome"[tiab]
  OR "gut microbiomes"[tiab]
  OR "gut microbiota"[tiab]
  OR "gut microflora"[tiab]
  OR "gut flora"[tiab]
  OR "intestinal microbiome"[tiab]
  OR "intestinal microbiomes"[tiab]
  OR "intestinal microbiota"[tiab]
  OR "intestinal microflora"[tiab]
  OR "intestinal flora"[tiab]
  OR "gastrointestinal microbiome"[tiab]
  OR "gastrointestinal microbiota"[tiab]
  OR "fecal microbiome"[tiab]
  OR "faecal microbiome"[tiab]
  OR "fecal microbiota"[tiab]
  OR "faecal microbiota"[tiab]
  OR "stool microbiome"[tiab]
  OR "stool microbiota"[tiab]
  OR "enteric microbiome"[tiab]
  OR "enteric microbiota"[tiab]
  OR "gut dysbiosis"[tiab]
  OR "intestinal dysbiosis"[tiab]
  OR "gut bacterial composition"[tiab]
  OR "intestinal bacterial composition"[tiab]
  OR (
    (microbiom*[tiab] OR microbiota[tiab] OR microflora[tiab] OR dysbiosis[tiab])
    AND
    (gut[tiab] OR intestin*[tiab] OR gastrointestinal[tiab] OR fecal[tiab] OR faecal[tiab] OR stool[tiab])
  )
  OR (
    (metagenom*[tiab] OR "16S rRNA"[tiab] OR "16S ribosomal RNA"[tiab])
    AND
    (gut[tiab] OR intestin*[tiab] OR gastrointestinal[tiab] OR fecal[tiab] OR faecal[tiab] OR stool[tiab])
  )
)
AND
(
  "Cerebral Small Vessel Diseases"[Mesh]
  OR "Leukoaraiosis"[Mesh]
  OR "cerebral small vessel disease"[tiab]
  OR "cerebral small vessel diseases"[tiab]
  OR "cerebral small-vessel disease"[tiab]
  OR "cerebral small-vessel diseases"[tiab]
  OR "cerebral small vascular disease"[tiab]
  OR "brain small vessel disease"[tiab]
  OR "brain small-vessel disease"[tiab]
  OR CSVD[tiab]
  OR cSVD[tiab]
  OR aCSVD[tiab]
  OR "cerebral microangiopathy"[tiab]
  OR "cerebral microangiopathies"[tiab]
  OR "cerebral microvascular disease"[tiab]
  OR "brain microvascular disease"[tiab]
  OR "arteriosclerotic cerebral small vessel disease"[tiab]
  OR "arteriolosclerotic cerebral small vessel disease"[tiab]
  OR "sporadic cerebral small vessel disease"[tiab]
  OR "age-related cerebral small vessel disease"[tiab]
  OR "subcortical ischemic vascular disease"[tiab]
  OR "subcortical ischaemic vascular disease"[tiab]
  OR "small vessel ischemic disease"[tiab]
  OR "small vessel ischaemic disease"[tiab]
  OR leukoaraios*[tiab]
  OR "white matter hyperintensity"[tiab]
  OR "white matter hyperintensities"[tiab]
  OR WMH[tiab]
  OR "white matter lesion"[tiab]
  OR "white matter lesions"[tiab]
```

```
  OR "white matter disease"[tiab]
  OR "white matter change"[tiab]
  OR "white matter changes"[tiab]
  OR Fazekas[tiab]
  OR lacune*[tiab]
  OR "lacunar lesion"[tiab]
  OR "lacunar lesions"[tiab]
  OR "lacunar infarct"[tiab]
  OR "lacunar infarcts"[tiab]
  OR "lacunar infarction"[tiab]
  OR "lacunar stroke"[tiab]
  OR "recent small subcortical infarct"[tiab]
  OR "recent small subcortical infarcts"[tiab]
  OR "small subcortical infarct"[tiab]
  OR "small subcortical infarcts"[tiab]
  OR "cerebral microbleed"[tiab]
  OR "cerebral microbleeds"[tiab]
  OR "brain microbleed"[tiab]
  OR "brain microbleeds"[tiab]
  OR "perivascular space"[tiab]
  OR "perivascular spaces"[tiab]
  OR "enlarged perivascular space"[tiab]
  OR "enlarged perivascular spaces"[tiab]
  OR "dilated perivascular space"[tiab]
  OR "dilated perivascular spaces"[tiab]
  OR "Virchow-Robin space"[tiab]
  OR "Virchow-Robin spaces"[tiab]
  OR "total small vessel disease burden"[tiab]
  OR "total CSVD burden"[tiab]
  OR "small vessel disease burden"[tiab]
  OR "small vessel disease score"[tiab]
  OR "peak width of skeletonized mean diffusivity"[tiab]
  OR PSMD[tiab]
  OR "Binswanger disease"[tiab]
  OR "Binswanger's disease"[tiab]
)
```

Table S1 — Embase.com exact executed strategy

```
(
  'gastrointestinal microbiome'/exp
  OR 'brain gut axis'/exp
  OR 'gut microbiome':ti,ab,kw
  OR 'gut microbiomes':ti,ab,kw
  OR 'gut microbiota':ti,ab,kw
  OR 'gut microflora':ti,ab,kw
  OR 'gut flora':ti,ab,kw
  OR 'intestinal microbiome':ti,ab,kw
  OR 'intestinal microbiomes':ti,ab,kw
  OR 'intestinal microbiota':ti,ab,kw
  OR 'intestinal microflora':ti,ab,kw
  OR 'intestinal flora':ti,ab,kw
  OR 'gastrointestinal microbiome':ti,ab,kw
  OR 'gastrointestinal microbiota':ti,ab,kw
  OR 'fecal microbiome':ti,ab,kw
  OR 'faecal microbiome':ti,ab,kw
  OR 'fecal microbiota':ti,ab,kw
  OR 'faecal microbiota':ti,ab,kw
  OR 'stool microbiome':ti,ab,kw
  OR 'stool microbiota':ti,ab,kw
  OR 'enteric microbiome':ti,ab,kw
  OR 'enteric microbiota':ti,ab,kw
  OR 'gut dysbiosis':ti,ab,kw
  OR 'intestinal dysbiosis':ti,ab,kw
  OR 'gut bacterial composition':ti,ab,kw
  OR 'intestinal bacterial composition':ti,ab,kw
  OR (((microbiom* OR microbiota OR microflora OR dysbiosis) NEAR/3
       (gut OR intestin* OR gastrointestinal OR fecal OR faecal OR stool)):ti,ab,kw)
  OR (((metagenom* OR '16S rRNA' OR '16S ribosomal RNA') NEAR/5
       (gut OR intestin* OR gastrointestinal OR fecal OR faecal OR stool)):ti,ab,kw)
)
AND
(
  'cerebral small vessel disease'/exp
  OR 'leukoaraiosis'/exp
  OR 'cerebral small vessel disease':ti,ab,kw
  OR 'cerebral small vessel diseases':ti,ab,kw
  OR 'cerebral small-vessel disease':ti,ab,kw
  OR 'cerebral small-vessel diseases':ti,ab,kw
  OR 'cerebral small vascular disease':ti,ab,kw
  OR 'brain small vessel disease':ti,ab,kw
  OR 'brain small-vessel disease':ti,ab,kw
  OR csvd:ti,ab,kw
  OR acsvd:ti,ab,kw
  OR 'cerebral microangiopathy':ti,ab,kw
  OR 'cerebral microangiopathies':ti,ab,kw
  OR 'cerebral microvascular disease':ti,ab,kw
  OR 'brain microvascular disease':ti,ab,kw
  OR 'arteriosclerotic cerebral small vessel disease':ti,ab,kw
  OR 'arteriolosclerotic cerebral small vessel disease':ti,ab,kw
  OR 'sporadic cerebral small vessel disease':ti,ab,kw
  OR 'age-related cerebral small vessel disease':ti,ab,kw
  OR 'subcortical ischemic vascular disease':ti,ab,kw
  OR 'subcortical ischaemic vascular disease':ti,ab,kw
```

```
  OR 'small vessel ischemic disease':ti,ab,kw
  OR 'small vessel ischaemic disease':ti,ab,kw
  OR leukoaraios*:ti,ab,kw
  OR 'white matter hyperintensity':ti,ab,kw
  OR 'white matter hyperintensities':ti,ab,kw
  OR wmh:ti,ab,kw
  OR 'white matter lesion':ti,ab,kw
  OR 'white matter lesions':ti,ab,kw
  OR 'white matter disease':ti,ab,kw
  OR 'white matter change':ti,ab,kw
  OR 'white matter changes':ti,ab,kw
  OR fazekas:ti,ab,kw
  OR lacune*:ti,ab,kw
  OR 'lacunar lesion':ti,ab,kw
  OR 'lacunar lesions':ti,ab,kw
  OR 'lacunar infarct':ti,ab,kw
  OR 'lacunar infarcts':ti,ab,kw
  OR 'lacunar infarction':ti,ab,kw
  OR 'lacunar stroke':ti,ab,kw
  OR 'recent small subcortical infarct':ti,ab,kw
  OR 'recent small subcortical infarcts':ti,ab,kw
  OR 'small subcortical infarct':ti,ab,kw
  OR 'small subcortical infarcts':ti,ab,kw
  OR 'cerebral microbleed':ti,ab,kw
  OR 'cerebral microbleeds':ti,ab,kw
  OR 'brain microbleed':ti,ab,kw
  OR 'brain microbleeds':ti,ab,kw
  OR 'perivascular space':ti,ab,kw
  OR 'perivascular spaces':ti,ab,kw
  OR 'enlarged perivascular space':ti,ab,kw
  OR 'enlarged perivascular spaces':ti,ab,kw
  OR 'dilated perivascular space':ti,ab,kw
  OR 'dilated perivascular spaces':ti,ab,kw
  OR 'virchow-robin space':ti,ab,kw
  OR 'virchow-robin spaces':ti,ab,kw
  OR 'total small vessel disease burden':ti,ab,kw
  OR 'total csvd burden':ti,ab,kw
  OR 'small vessel disease burden':ti,ab,kw
  OR 'small vessel disease score':ti,ab,kw
  OR 'peak width of skeletonized mean diffusivity':ti,ab,kw
  OR psmd:ti,ab,kw
  OR binswanger*:ti,ab,kw
)
```

Table S1 — Scopus exact executed strategy

```
TITLE-ABS-KEY(
  "gut microbiome"
  OR "gut microbiomes"
  OR "gut microbiota"
  OR "gut microflora"
  OR "gut flora"
  OR "intestinal microbiome"
  OR "intestinal microbiomes"
  OR "intestinal microbiota"
  OR "intestinal microflora"
  OR "intestinal flora"
  OR "gastrointestinal microbiome"
  OR "gastrointestinal microbiota"
  OR "fecal microbiome"
  OR "faecal microbiome"
  OR "fecal microbiota"
  OR "faecal microbiota"
  OR "stool microbiome"
  OR "stool microbiota"
  OR "enteric microbiome"
  OR "enteric microbiota"
  OR "gut dysbiosis"
  OR "intestinal dysbiosis"
  OR "gut bacterial composition"
  OR "intestinal bacterial composition"
  OR ((microbiom* OR microbiota OR microflora OR dysbiosis) W/3
      (gut OR intestin* OR gastrointestinal OR fecal OR faecal OR stool))
  OR ((metagenom* OR "16S rRNA" OR "16S ribosomal RNA") W/5
      (gut OR intestin* OR gastrointestinal OR fecal OR faecal OR stool))
)
AND
TITLE-ABS-KEY(
  "cerebral small vessel disease"
  OR "cerebral small vessel diseases"
  OR "cerebral small-vessel disease"
  OR "cerebral small-vessel diseases"
  OR "cerebral small vascular disease"
  OR "brain small vessel disease"
  OR "brain small-vessel disease"
  OR CSVD
  OR aCSVD
  OR "cerebral microangiopathy"
  OR "cerebral microangiopathies"
  OR "cerebral microvascular disease"
  OR "brain microvascular disease"
  OR "arteriosclerotic cerebral small vessel disease"
  OR "arteriolosclerotic cerebral small vessel disease"
  OR "sporadic cerebral small vessel disease"
```

```
  OR "age-related cerebral small vessel disease"
  OR "subcortical ischemic vascular disease"
  OR "subcortical ischaemic vascular disease"
  OR "small vessel ischemic disease"
  OR "small vessel ischaemic disease"
  OR leukoaraios*
  OR "white matter hyperintensity"
  OR "white matter hyperintensities"
  OR WMH
  OR "white matter lesion"
  OR "white matter lesions"
  OR "white matter disease"
  OR "white matter change"
  OR "white matter changes"
  OR Fazekas
  OR lacune*
  OR "lacunar lesion"
  OR "lacunar lesions"
  OR "lacunar infarct"
  OR "lacunar infarcts"
  OR "lacunar infarction"
  OR "lacunar stroke"
  OR "recent small subcortical infarct"
  OR "recent small subcortical infarcts"
  OR "small subcortical infarct"
  OR "small subcortical infarcts"
  OR "cerebral microbleed"
  OR "cerebral microbleeds"
  OR "brain microbleed"
  OR "brain microbleeds"
  OR "perivascular space"
  OR "perivascular spaces"
  OR "enlarged perivascular space"
  OR "enlarged perivascular spaces"
  OR "dilated perivascular space"
  OR "dilated perivascular spaces"
  OR "Virchow-Robin space"
  OR "Virchow-Robin spaces"
  OR "total small vessel disease burden"
  OR "total CSVD burden"
  OR "small vessel disease burden"
  OR "small vessel disease score"
  OR "peak width of skeletonized mean diffusivity"
  OR PSMD
  OR "Binswanger disease"
  OR "Binswanger's disease"
)
```

Table S1 — Web of Science Core Collection exact executed strategy

```
TS=(
  "gut microbiome"
  OR "gut microbiomes"
  OR "gut microbiota"
  OR "gut microflora"
  OR "gut flora"
  OR "intestinal microbiome"
  OR "intestinal microbiomes"
  OR "intestinal microbiota"
  OR "intestinal microflora"
  OR "intestinal flora"
  OR "gastrointestinal microbiome"
  OR "gastrointestinal microbiota"
  OR "fecal microbiome"
  OR "faecal microbiome"
  OR "fecal microbiota"
  OR "faecal microbiota"
  OR "stool microbiome"
  OR "stool microbiota"
  OR "enteric microbiome"
  OR "enteric microbiota"
  OR "gut dysbiosis"
  OR "intestinal dysbiosis"
  OR "gut bacterial composition"
  OR "intestinal bacterial composition"
  OR ((microbiom* OR microbiota OR microflora OR dysbiosis) NEAR/3
      (gut OR intestin* OR gastrointestinal OR fecal OR faecal OR stool))
  OR ((metagenom* OR "16S rRNA" OR "16S ribosomal RNA") NEAR/5
      (gut OR intestin* OR gastrointestinal OR fecal OR faecal OR stool))
)
AND
TS=(
  "cerebral small vessel disease"
  OR "cerebral small vessel diseases"
  OR "cerebral small-vessel disease"
  OR "cerebral small-vessel diseases"
  OR "cerebral small vascular disease"
  OR "brain small vessel disease"
  OR "brain small-vessel disease"
  OR CSVD
  OR aCSVD
  OR "cerebral microangiopathy"
  OR "cerebral microangiopathies"
  OR "cerebral microvascular disease"
```

```
  OR "brain microvascular disease"
  OR "arteriosclerotic cerebral small vessel disease"
  OR "arteriolosclerotic cerebral small vessel disease"
  OR "sporadic cerebral small vessel disease"
  OR "age-related cerebral small vessel disease"
  OR "subcortical ischemic vascular disease"
  OR "subcortical ischaemic vascular disease"
  OR "small vessel ischemic disease"
  OR "small vessel ischaemic disease"
  OR leukoaraios*
  OR "white matter hyperintensity"
  OR "white matter hyperintensities"
  OR WMH
  OR "white matter lesion"
  OR "white matter lesions"
  OR "white matter disease"
  OR "white matter change"
  OR "white matter changes"
  OR Fazekas
  OR lacune*
  OR "lacunar lesion"
  OR "lacunar lesions"
  OR "lacunar infarct"
  OR "lacunar infarcts"
  OR "lacunar infarction"
  OR "lacunar stroke"
  OR "recent small subcortical infarct"
  OR "recent small subcortical infarcts"
  OR "small subcortical infarct"
  OR "small subcortical infarcts"
  OR "cerebral microbleed"
  OR "cerebral microbleeds"
  OR "brain microbleed"
  OR "brain microbleeds"
  OR "perivascular space"
  OR "perivascular spaces"
  OR "enlarged perivascular space"
  OR "enlarged perivascular spaces"
  OR "dilated perivascular space"
  OR "dilated perivascular spaces"
  OR "Virchow-Robin space"
  OR "Virchow-Robin spaces"
  OR "total small vessel disease burden"
  OR "total CSVD burden"
  OR "small vessel disease burden"
  OR "small vessel disease score"
  OR "peak width of skeletonized mean diffusivity"
  OR PSMD
  OR "Binswanger disease"
  OR "Binswanger's disease"
)
```

Table S2. Report-level full-text eligibility assessment, exclusions, and related reports

No.	Report title	Final decision	Code	Final reason	Cohort/report family	Source
1	Cerebral small vessel disease burden is associated with decreased abundance of gut Barnesiella intestinihominis bacterium in the Framingham Heart Study	Included		Included in the qualitative synthesis.	Framingham Heart Study	https://www.nature.com/articles/s41598-023-40872-5
2	Cerebral Small Vessel Disease Burden is Associated with Decreased Abundance of Gut Barnesiella intestinihominis Bacterium in the Framingham Heart Study	Related/superseded report	REL	Preprint of candidate 1; superseded by the peer-reviewed article.	Framingham Heart Study	https://doi.org/10.1101/2022.09.27.509283
3	The gut microbiome is associated with markers of cerebral small vessel disease	Related/superseded report	REL	Conference precursor; no unique sample or outcome beyond the later full article.	Framingham Heart Study	https://doi.org/10.1002/alz.067130
4	Relationship Between Plasma Lipopolysaccharides, Gut Microbiota, and Dementia: A Cross-Sectional Study	Excluded	EX09	No direct microbiome–MRI/CSVD association.	GIMLET / Japanese memory-clinic cohort	https://pubmed.ncbi.nlm.nih.gov/35213381/
5	Relationship Between Plasma Lipopolysaccharides, Gut Microbiota, and Dementia: A Cross-Sectional Study	Related/superseded report	REL	Alternate/republication record for candidate 4; no unique eligible evidence.	GIMLET / Japanese memory-clinic cohort	https://doi.org/10.3233/aiad220035
6	Relationship Between Plasma Neurofilament Light Chain, Gut Microbiota, and Dementia: A Cross-Sectional Study	Excluded	EX09	No direct microbiome–MRI/CSVD association.	GIMLET / Japanese memory-clinic cohort	https://pubmed.ncbi.nlm.nih.gov/35180112/
7	Relationship Between Plasma Neurofilament Light Chain, Gut Microbiota, and Dementia: A Cross-Sectional Study	Related/superseded report	REL	Alternate/republication record for candidate 6; no unique eligible evidence.	GIMLET / Japanese memory-clinic cohort	https://doi.org/10.3233/aiad220033
8	The Association between Cerebral Small Vessel Disease and the Gut Microbiome: A Cross-Sectional Analysis	Included		Included in the qualitative synthesis.	GIMLET / Japanese memory-clinic cohort	https://doi.org/10.1016/j.jstrokecerebrovasdis.2020.105568
9	Gut microbiota and dementia: A cross-sectional study	Related/superseded report	REL	Conference precursor; no unique extractable CSVD result beyond later full reports.	GIMLET / Japanese memory-clinic cohort	https://doi.org/10.1161/str.50.suppl_1.wp569
10	The relationship between the gut microbiome and mild cognitive impairment in patients without dementia: A cross-sectional study conducted in Japan	Related/superseded report	REL	Conference precursor of candidate 11 with the same MCI/microbiome analysis.	GIMLET / Japanese memory-clinic cohort	https://doi.org/10.1016/j.jns.2019.10.789
11	The relationship between the gut microbiome and mild cognitive impairment in patients without dementia: a cross-sectional study conducted in Japan	Included		Included in the qualitative synthesis.	GIMLET / Japanese memory-clinic cohort	https://www.nature.com/articles/s41598-019-55851-y
12	GUT DYSBIOSIS AND CEREBRAL SMALL VESSEL DISEASE: A MULTIMODAL STUDY	Excluded	EX10	Conference abstract only; no full-text report identified; insufficient detail for reliable extraction/appraisal.	Hong Kong middle-aged MRI–microbiome cohort	https://research.cuhk.edu.hk/en/publications/gut-dysbiosis-and-cerebral-small-vessel-disease-a-multimodal-stud/
13	SEX-SPECIFIC ASSOCIATIONS IN GUT MICROBIOTA DYSBIOSIS AND CEREBRAL SMALL VESSEL DISEASE IN MIDDLE-AGED CHINESE: A METAGENOMIC SEQUENCING ANALYSIS	Excluded	EX10	Conference abstract only; no full-text report identified; insufficient detail for reliable extraction/appraisal.	Hong Kong middle-aged MRI–microbiome cohort	https://doi.org/10.1177/23969873241245672
14	Early-stage white matter hyperintensities (WMH) associated with shotgun gut microbiota species among healthy aging adults	Excluded	EX10	Conference abstract only; no full-text report identified; insufficient detail for reliable extraction/appraisal.	Hong Kong middle-aged MRI–microbiome cohort	https://karger.com/ced/article-pdf/52/Suppl.%202/6/4052750/000534859.pdf
15	Klotho in the gut-cerebrovascular axis: Sex-linked gut microbiome associations with stroke risk and small vessel disease	Excluded	EX10	Conference abstract only; no full-text report identified; insufficient detail for reliable extraction/appraisal.	Hong Kong middle-aged MRI–microbiome cohort	https://www.hkmj.org/system/files/2023-S5.pdf
16	Clostridium shows a higher abundance in less neurovascular and neurodegenerative aspects: A microbiomewide association study	Excluded	EX10	Conference abstract only; no full-text report identified; insufficient detail for reliable extraction/appraisal.	Rotterdam Study	https://alz-journals.onlinelibrary.wiley.com/doi/abs/10.1002/alz.044743

No.	Report title	Final decision	Code	Final reason	Cohort/report family	Source
17	Association between gut microbiota and imaging biomarkers in arteriosclerotic cerebral small vessel disease with idiopathic normal pressure hydrocephalus	Included		Included in the qualitative synthesis.	aCSVD–iNPH cohort	https://www.frontiersin.org/journals/neuroscience/articles/10.3389/fnins.2026.1789687/full
18	Correlation study between gut microbiota and intestinal permeability in cerebral small vessel disease	Included		Included in the qualitative synthesis.	CSVD-CI intestinal-permeability cohort	https://pubmed.ncbi.nlm.nih.gov/41761646/
19	Resolving cognitive heterogeneity in white matter hyperintensities through integrated analyses of microbiome, metabolome, and brain glymphatic function	Included		Included in the qualitative synthesis.	Anhui WMH multimodal cohort	https://pubmed.ncbi.nlm.nih.gov/41700076/
20	Characterizing the role of the microbiota-gut-brain axis in cerebral small vessel disease: An integrative multi-omics study	Included		Included in the qualitative synthesis.	Anhui WMH multimodal cohort	https://pubmed.ncbi.nlm.nih.gov/39505226/
21	Mediation of Regional Cerebral Blood Flow in the Relationship between Specific Gut Microbiota and Cognition in Vascular Cognitive Impairment	Excluded	EX05	No conventional MRI-defined CSVD marker or separable sporadic CSVD phenotype.	Chinese Imaging, Biomarkers and Lifestyle VCI cohort	https://pubmed.ncbi.nlm.nih.gov/38108351/
22	Multi-omics analysis of gut-brain axis reveals novel microbial and neurotransmitter signatures in patients with arteriosclerotic cerebral small vessel disease	Included		Included in the qualitative synthesis.	Guangzhou aCSVD immune/multi-omics cohort	https://www.sciencedirect.com/science/article/pii/S104366182400330X
23	The association between alpha diversity of gut microbiota, neuroimaging markers and cognitive function in cerebral small vessel disease	Included		Included in the qualitative synthesis.	Anhui CSVD cognition/neurovascular cohort	https://pubmed.ncbi.nlm.nih.gov/38215865/
24	The gut microbial signatures of patients with lacunar cerebral infarction	Excluded	EX06	Acute lacunar infarction only; no separable chronic MRI-defined CSVD marker.	Acute lacunar infarction cohort	https://pubmed.ncbi.nlm.nih.gov/37538045/
25	The influence of alterations in the composition of intestinal microbiota on neurovascular coupling and cognitive dysfunction in individuals afflicted with CSVD	Included		Included in the qualitative synthesis.	Anhui CSVD cognition/neurovascular cohort	https://pubmed.ncbi.nlm.nih.gov/39510273/
26	Alteration and clinical potential in gut microbiota in patients with cerebral small vessel disease	Included		Included in the qualitative synthesis.	Shi et al. CSVD diagnostic cohort	https://www.frontiersin.org/journals/cellular-and-infection-microbiology/articles/10.3389/fcimb.2023.1231541/full
27	Gut microbiota from patients with arteriosclerotic CSVD induces higher IL-17A production in neutrophils via activating RORγt	Included		Included in the qualitative synthesis.	Guangzhou aCSVD immune/multi-omics cohort	https://www.science.org/doi/10.1126/sciadv.abe4827

Codes: EX05, no eligible MRI-defined CSVD outcome; EX06, stroke-only outcome without separable chronic CSVD; EX09, no direct microbiome–MRI/CSVD association; EX10, abstract-only conference report without an identifiable full publication; REL, related or superseded report.

Table S3. Cohort-overlap classification matrix

Report(s)	Recruitment centre / framework	Recruitment period	Sample size / group structure	Authorship / ethics information	Classification	Basis for classification
Fongang et al., 2023 [21]	Framingham Heart Study, USA	FHS examination 3, 2016-2019	N=972; population-based sample with stool, MRI, and cognition	No related included report from the same study framework identified	Provisionally independent	No evidence of participant overlap with another included report.
Saji et al., 2019 [22]; Saji et al., 2021 [30]	NCGG memory clinic, Japan; GIMLET study	Parent GIMLET cohort: Mar 2016-Mar 2017	2019: N=82 (MCI 61, normal cognition 21); 2021: N=87 (MCI 64, non-MCI 23)	Same GIMLET/NCGG framework and essentially identical author group	Probable overlap / same cohort family	Same GIMLET framework and recruitment period; closely related samples; not treated as independent replication.
Cai et al., 2026 [23]	Maoming People's Hospital, China	Aug 2020-Nov 2024	N=95; aCSVD 45, aCSVD-iNPH 20, HC 30	No related included report from the same centre identified	Provisionally independent	No evidence of shared participants with another included report.
Xu et al., 2026 [24]	First Affiliated Hospital of Anhui Medical University, China	Aug 2018-Mar 2022	N=145; HC 56, WMH-NC 40, WMH-CI 49	Shared investigators with other Anhui reports	Probable overlap within Anhui family	Same centre, overlapping recruitment periods, related participant definitions and investigators; participant-level overlap not reported.
Song et al., 2024 [25]	First Affiliated Hospital of Anhui Medical University, China	Aug 2020-Jul 2022	N=83; CSVD 37, HC 46	Shared investigators with other Anhui reports	Probable overlap within Anhui family	Same centre and overlapping recruitment windows; participant-level overlap unresolved.
Zhang et al., 2024 [27]	First Affiliated Hospital of Anhui Medical University, China	Aug 2020-Aug 2022	N=144; HC 40, CSVD-NCI 61, CSVD-MCI 43	Ethics approval PJ 2022-09-09; substantial investigator overlap with Huang et al. [31]	Potential overlap with Huang et al. [31]; related Anhui family	Same centre and recruitment period, identical HC sample size, related group structure, overlapping investigators, and same ethics approval; exact overlap not reported.
Huang et al., 2024 [31]	First Affiliated Hospital of Anhui Medical University, China	Aug 2020-Feb 2022	N=118; HC 40, CSVD-NCI 43, CSVD-MCI 35	Ethics approval PJ 2022-09-09; substantial investigator overlap with Zhang et al. [27]	Potential overlap with Zhang et al. [27]; related Anhui family	Recruitment nested within Zhang et al.; same centre, HC sample size, related group structure, investigators, and ethics approval; exact overlap not reported.
Huang et al., 2024 [26]; Cai et al., 2021 [29]	Third Affiliated Hospital of Sun Yat-sen University, China	Huang: Sep 2020-Oct 2021; Cai: Jul 2018-Aug 2019	Huang: N=67 (aCSVD 37, HC 30); Cai: N=117 (aCSVD 55, HC 62)	Shared institution and several investigators	Provisionally independent / separate cohort families	Non-overlapping documented recruitment windows; no evidence of shared participants.
Shi et al., 2023 [28]	Wuxi People's Hospital, China	Not reported	N=82; CSVD 64, community HC 18	No related included report from the same centre identified	Provisionally independent	No evidence of participant overlap with another included report.
Chen et al., 2026 [32]	Affiliated Hospital of Guangdong Medical University, China	Mar 2019-Jul 2020	N=41; CSVD-CI 21, HC 20	No related included report from the same centre identified	Provisionally independent	No evidence of participant overlap with another included report.

Cohort relationships were classified conservatively on the basis of recruitment centre and period, sample size and group structure, authorship overlap, and ethics information where available. “Probable overlap” indicates that several features strongly suggested shared recruitment, whereas “potential overlap” indicates that overlap could not be excluded but could not be established from the published information. “Provisionally independent” indicates that no evidence of shared participants was identified; it does not establish complete cohort independence. Reference numbers correspond to the reference list of the main manuscript.

Table S4. Detailed study characteristics, microbiome methods, and MRI phenotypes

Table S4A. Study and cohort characteristics

ID	Report	Setting	Design	Recruitment	Population	Groups	N	Age	Sex	Cohort relationship	DOI
1	**Fongang et al., 2023**	USA; Framingham Heart Study	Population-based cohort, cross-sectional secondary analysis	FHS examination 3, 2016–2019	972 participants with stool, MRI and cognition; dementia and incident stroke excluded	No case/control groups	972	Median 54 years (IQR 47–60)	540 women (56%)	Provisionally independent Framingham	10.1038/s41598-023-40872-5
2	**Saji et al., 2019**	Japan; NCGG memory clinic, GIMLET study	Hospital-based cross-sectional sub-study	Not stated in this paper; parent GIMLET report recruited Mar 2016–Mar 2017	Patients without dementia; 61 MCI and 21 normal cognition	MCI 61; normal cognition 21	82	Median 76 years (IQR 68–80)	43 women (52.4%)	GIMLET overlap / same cohort family with study 10	10.1038/s41598-019-55851-y
3	**Cai et al., 2026**	China; Maoming People's Hospital	Single-centre cross-sectional three-group study	Aug 2020–Nov 2024	aCSVD, aCSVD-iNPH and elderly healthy controls	aCSVD 45; aCSVD-iNPH 20; HC 30	95	Age ≥60 by eligibility criteria	See Table 1	Provisionally independent Maoming	10.3389/fnins.2026.1789687
4	**Xu et al., 2026**	China; First Affiliated Hospital of Anhui Medical University	Single-centre cross-sectional three-group multi-omics study	Aug 2018–Mar 2022	Chinese Han adults with moderate–severe WMH, stratified by cognition, plus controls	HC 56; WMH-NC 40; WMH-CI 49	145	HC 61.5±7.5; WMH-NC 63.8±6.8; WMH-CI 64.9±7.9	Women: 30/56, 14/40, 24/49	Probable Anhui overlap	10.1002/alz.71201
5	**Song et al., 2024**	China; First Affiliated Hospital of Anhui Medical University	Single-centre cross-sectional case-control multi-omics study	Aug 2020–Jul 2022	CSVD patients selected for WMH phenotype and matched healthy controls	CSVD 37; HC 46	83	Age 50–80 by eligibility criteria	Age- and sex-matched	Probable Anhui overlap	10.1016/j.neuroimage.2024.120918
6	**Huang et al., 2024**	China; Third Affiliated Hospital of Sun Yat-Sen University	Single-centre cross-sectional case-control shotgun multi-omics study	Sep 2020–Oct 2021	aCSVD patients and healthy controls	aCSVD 37; HC 30	67	aCSVD 67.7±7.2; HC 64.8±5.3	See Table 1	Provisionally independent; separate Sun Yat-sen cohort	10.1016/j.phrs.2024.107385
7	**Zhang et al., 2024**	China; First Affiliated Hospital of Anhui Medical University	Single-centre cross-sectional three-group study	Aug 2020–Aug 2022	Newly diagnosed CSVD, stratified by cognition, and matched healthy controls	CSVD-NCI 61; CSVD-MCI 43; HC 40	144	HC 63.0±5.7; CSVD 64.8±8.3	HC 21/19; CSVD 61/43 male/female	Potential Anhui overlap with study 11	10.1016/j.brainresbull.2024.111115
8	**Shi et al., 2023**	China; Wuxi People's Hospital	Single-centre cross-sectional case-control study	Recruitment period not stated in main PDF	Chinese Han CSVD patients and community controls	CSVD 64; HC 18	82	CSVD 69.2±5.7; HC 66.2±5.5	CSVD 32/32; HC 7/11 male/female	Provisionally independent Wuxi	10.3389/fcimb.2023.1231541
9	**Cai et al., 2021**	China; Third Affiliated Hospital of Sun Yat-Sen University	Single-centre cross-sectional human cohort plus mechanistic experiments	Jul 2018–Aug 2019	aCSVD patients and age-matched healthy controls	aCSVD 55 (burden 0–1: 30; burden 2–4: 25); HC 62	117	HC 64.8±9.4; patient age in supplement	HC 33 men (reported as 60%); patient sex in supplement	Provisionally independent; separate Sun Yat-sen cohort	10.1126/sciadv.abe4827
10	**Saji et al., 2021**	Japan; NCGG memory clinic, GIMLET study	Hospital-based cross-sectional sub-analysis of prospective cohort	Mar 2016–Mar 2017	Patients without dementia or stroke history	MCI 64; non-MCI 23	87	Median 76 years (IQR 68–81)	43 women (49.4%)	GIMLET overlap / same cohort family with study 2	10.1016/j.jstrokecerebrovasdis.2020.105568
11	**Huang et al., 2024**	China; First Affiliated Hospital of Anhui Medical University	Single-centre cross-sectional three-group study	Aug 2020–Feb 2022	CSVD stratified by cognition and matched healthy controls	CSVD-NCI 43; CSVD-MCI 35; HC 40	118	HC median 62.5; CSVD median 65.0	HC 20/20; CSVD 44/34 male/female	Potential Anhui overlap with study 7	10.1016/j.brainres.2024.148757
12	**Chen et al., 2026**	China; Affiliated Hospital of Guangdong Medical University	Single-centre cross-sectional case-control human study plus FMT mouse experiment	Mar 2019–Jul 2020	CSVD with cognitive impairment and healthy controls	CSVD-CI 21; HC 20	41	CSVD-CI 68.4±9.6; HC 65.1±7.9	Men 16/21 vs 13/20	Provisionally independent Guangdong	10.1177/13872877261418554

Table S4B. Microbiome and MRI methodology

ID	Report	Stool collection/storage	Assay	Platform	Bioinformatics/diversity	Microbiome statistics	MRI acquisition	MRI/CSVD outcomes	Adjustment/modelling
1	**Fongang et al., 2023**	Stool in 100% ethanol; −80°C	16S rRNA V4	Illumina MiSeq, 2×150 bp	QIIME2; OTU clustering 97%; Greengenes; MaAsLin2; negative-binomial models	Differential abundance and multivariable continuous association	1.5/3T; T1, FLAIR, DTI	WMH volume (%ICV), PSMD, executive function	Age, age², sex, BMI, education (EF), MRI–stool time
2	**Saji et al., 2019**	Home collection; frozen and stored at −81°C in NCGG Biobank	16S-based T-RFLP; 10 bacterial groups and enterotypes	TechnoSuruga Laboratory	T-RFLP; enterotype I *Bacteroides* >30%, II *Prevotella* >15%, III other; F/B ratio	Group comparisons and multivariable logistic regression	Brain MRI; protocol in methods/supplement	WMH, silent lacunar infarct, CMB; VSRAD	Age, sex, education, risk factors and MRI variables depending on model
3	**Cai et al., 2026**	Fresh feces; standard DNA extraction	16S rRNA V3–V4	Illumina MiSeq or NovaSeq 6000	Alpha: Shannon, Simpson, Chao1, observed OTU, Pielou; beta: Jaccard/Bray-Curtis PCoA/PERMANOVA; LEfSe	Kruskal–Wallis, FDR pairwise comparisons, Spearman correlations	MRI per STRIVE; blinded neuroradiologist scoring	Lacunes, WMH score, CMB count, deep medullary vein score	Primarily unadjusted correlations; FDR correction
4	**Xu et al., 2026**	Within 1 day of MRI; −80°C	16S rDNA V4	Illumina NovaSeq, 250-bp paired end	UNOISE3 ASVs; RDP chimera reference; relative abundance; PERMANOVA; Dunn/Bonferroni	Kruskal–Wallis/ANOVA, post hoc correction, correlations and mediation	3T structural, diffusion and resting-state fMRI	WMH Fazekas/volume; CPV, PVS volume fraction, free water, ALPS, BOLD-CSF coupling	Multi-stage correlation/mediation; confounder handling varies by analysis
5	**Song et al., 2024**	Within 1 day of MRI; aliquoted and immediately −80°C	16S rRNA V4	Illumina NovaSeq, 250-bp paired end	UNOISE3 ASVs; RDP reference; LEfSe; multi-omics correlation with FDR	LEfSe and FDR-corrected multi-stage correlations	3T multimodal MRI	WMH phenotype; GMV/cortical thickness, DTI, rs-fMRI and network measures	Group tests plus FDR-corrected correlation networks

ID	Report	Stool collection/storage	Assay	Platform	Bioinformatics/diversity	Microbiome statistics	MRI acquisition	MRI/CSVD outcomes	Adjustment/modelling
6	**Huang et al., 2024**	≈1 g stool in DNA protective solution; −80°C	Shotgun metagenomics	Illumina NovaSeq 6000, 150-bp paired end	Quality control, assembly/binning, Kraken2 taxonomic profiling, LEfSe, pathway analysis	Wilcoxon/chi-square; PCoA Bray-Curtis (adonis $R^2 = 0.08$, $p = 0.001$)	MRI in aCSVD patients; details partly in supplement	Clinical aCSVD status and total CSVD burden	Main case-control analyses; limited adjusted modelling
7	**Zhang et al., 2024**	Collected within 1 day of MRI; −80°C	16S rDNA V4	Illumina NovaSeq, 250-bp paired end	QIIME 1.9.1; UPARSE 97% OTU; UCHIME; SILVA 138; rarefaction; LEfSe; Bray-Curtis	Kruskal–Wallis/Bonferroni; partial correlations adjusted for age, sex, BMI	3T; structural MRI, ASL and BOLD	WMH volume, lacunes, CMB, total burden; CBF/mReHo, CBF/mfALFF, CBF/mALFF, CBF/mDC	Age/sex/BMI in partial correlations; group comparisons largely unadjusted
8	**Shi et al., 2023**	Fasting morning sample with DNA stabilizer; −80°C	16S rRNA sequencing; detailed region/platform in supplement	Sequencing by Genesky Biotechnologies	QIIME2; alpha indices; PLS-DA; LEfSe; PICRUSt2; LASSO	t/Mann–Whitney/chi-square; Pearson correlations; LASSO ROC	Structural and resting-state functional MRI	Fazekas, lacunes, CMB, EPVS, atrophy; ALFF brain regions	Primarily unadjusted correlations; multiple testing details in supplement
9	**Cai et al., 2021**	Fecal DNA extraction kit	16S rRNA V3–V4	Illumina MiSeq	QIIME2 v2017.12; 99% OTUs; Greengenes 13_8; Chao1/observed OTU/Pielou/Faith PD/Shannon/Simpson; LEfSe	ANOVA, Spearman, ROC; burden-stratified analyses	3T MRI	Lacunes, d-WMH, p-WMH, lobar/deep CMB, PVS, atrophy, total aCSVD burden	Mostly unadjusted discovery analyses; ROC includes epidemiological factors
10	**Saji et al., 2021**	Home collection; frozen and stored at −81°C	16S-based T-RFLP and enterotypes	TechnoSuruga Laboratory	T-RFLP 10 groups; enterotypes; fecal metabolites	Univariate and adjusted logistic regression; ordinal SVD models	1.5T MRI	SLI, WMH (Fazekas), CMB, EPVS; total SVD score 0–4; VSRAD	Adjusted for age, sex, education and vascular risk factors
11	**Huang et al., 2024**	Within 1 day of MRI; −80°C	16S rDNA V4	Illumina NovaSeq, 250-bp paired end	QIIME 1.9.1; UPARSE 97% OTU; UCHIME/SILVA; MOTHUR/QIIME alpha diversity	Negative-binomial and linear regression; mediation	3T structural MRI	WMH volume/Fazekas, lacune count, CMB count, total CSVD burden (EPVS omitted)	Models adjusted for age/sex and vascular risk factors
12	**Chen et al., 2026**	Human stool stored at −80°C within 1 hour	16S rRNA sequencing; human region/platform not explicit in main PDF	Sequencing by Shanghai Meiji; mouse V3–V4 on MiSeq	Majorbio platform; Sobs/Chao/Ace/Shannon/Simpson; PLS-DA; LEfSe; MaAsLin for permeability	t/Wilcoxon/chi-square; Spearman; random forest and MaAsLin	WMH rated by Fazekas	Periventricular and deep WMH summed 0–6	Small unadjusted case-control study; human and mouse analyses must remain separate

Table S5. Alpha- and beta-diversity findings

Rows retain the original clinical contrast and reporting scale. Overlapping Anhui reports were not counted as independent replication.

ID	Report	Cohort/independence	Contrast	Metric	Disease/high-burden	Comparator/low-burden	P value	Direction	Scale/format	Synthesis role	Limitation	Source
1	Fongang et al., 2023	Provisionally independent Framingham	Continuous CSVD burden association	Multiple alpha indices	No significant alpha-diversity association	—	NS	Null	Continuous association	Narrative	Continuous population-based outcome; not case-control.	Main + supplementary SF1/SF2; https://doi.org/10.1038/s41598-023-40872-5
3	Cai et al., 2026	Provisionally independent Maoming	aCSVD / aCSVD-iNPH / HC	Multiple alpha indices	No significant group difference reported	No significant group difference reported	NS	No extractable direction	Narrative/figure	Narrative	No compatible numeric summary or reliable direction.	Main + Supplementary Table S1; https://doi.org/10.3389/fnins.2026.1789687
6	Huang et al., 2024 multi-omics	Provisionally independent Sun Yat-sen 2020–2021	aCSVD vs HC	Chao1	Lower in aCSVD (violin plot only)	Higher in HC	0.0027	Lower in CSVD	Figure	Directional evidence	Exact group summary statistics not reported.	Main Fig. 1A; https://doi.org/10.1016/j.phrs.2024.107385
6	Huang et al., 2024 multi-omics	Provisionally independent Sun Yat-sen 2020–2021	aCSVD vs HC	Shannon	Lower in aCSVD (violin plot only)	Higher in HC	0.0053	Lower in CSVD	Figure	Directional evidence	Exact group summary statistics not reported.	Main Fig. 1A; https://doi.org/10.1016/j.phrs.2024.107385
7	Zhang et al., 2024	Anhui family; overlaps/nests study 11	CSVD vs HC	Chao1	993.86 (737.79)	1196.12 (748.16)	0.036	Lower in CSVD	Single-parenthesis summary	Secondary Anhui evidence	Non-parametric analysis; spread measure not defined.	Main Table 1; https://doi.org/10.1016/j.brainresbull.2024.111115
7	Zhang et al., 2024	Anhui family; overlaps/nests study 11	CSVD vs HC	Shannon	5.93 (0.98)	5.69 (1.80)	0.371	Slightly higher in CSVD; NS	Single-parenthesis summary	Secondary Anhui evidence	Null result; overlaps study 11.	Main Table 1; https://doi.org/10.1016/j.brainresbull.2024.111115
8	Shi et al., 2023	Provisionally independent Wuxi	CSVD vs HC	Chao1	Lower in CSVD (boxplot)	Higher in HC	0.077	Lower in CSVD	Supplementary boxplot	Directional evidence	Trend only; exact summary statistics not reported.	Supplementary Fig. 2A; https://doi.org/10.3389/fcimb.2023.1231541
8	Shi et al., 2023	Provisionally independent Wuxi	CSVD vs HC	Shannon	Lower in CSVD (boxplot)	Higher in HC	0.033	Lower in CSVD	Supplementary boxplot	Directional evidence	Significant; exact summary statistics not reported.	Supplementary Fig. 2A; https://doi.org/10.3389/fcimb.2023.1231541
9	Cai et al., 2021	Provisionally independent Sun Yat-sen 2018–2019	Burden 2–4 vs burden 0–1 within aCSVD	Chao1	Higher in burden 2–4	Lower in burden 0–1	<0.05	Higher with greater burden	Within-CSVD contrast	Within-CSVD contrast	Within-CSVD severity contrast; not comparable with case-control contrasts.	Main + Supplement; https://doi.org/10.1126/sciadv.abe4827
11	Huang et al., 2024 alpha	Anhui family; preferred alpha report	CSVD vs HC	Chao1	Raw median 839.43 (657.19,1292.47); ln mean±SD 6.84±0.40, n=78	Raw median 1337.53 (928.77,1676.93); ln mean±SD 7.13±0.40, n=40	<0.001	Lower in CSVD	Raw median (IQR) + ln mean±SD	Primary Anhui evidence	ln-transformed moments reported; preferred alpha estimate in this family.	Main Table 1; https://doi.org/10.1016/j.brainres.2024.148757
11	Huang et al., 2024 alpha	Anhui family; preferred alpha report	CSVD vs HC	Shannon	Raw median 5.79 (5.24,6.22); ln mean±SD 1.71±0.20, n=78	Raw median 5.70 (4.62,6.31); ln mean±SD 1.68±0.29, n=40	0.493 (ln); 0.712 raw	Null	Raw median (IQR) + ln mean±SD	Primary Anhui evidence	No evidence of lower Shannon diversity.	Main Table 1; https://doi.org/10.1016/j.brainres.2024.148757
12	Chen et al., 2026	Provisionally independent Guangdong	CSVD-CI vs HC	Chao	247.02±90.21, n=21	295.82±109.13, n=20	0.32	Lower in CSVD-CI	Raw mean±SD	Primary evidence	Small sample; CSVD-CI only.	Main alpha-diversity table; https://doi.org/10.1177/13872877261418554
12	Chen et al., 2026	Provisionally independent Guangdong	CSVD-CI vs HC	Shannon	2.88±0.62, n=21	3.07±0.71, n=20	0.24	Lower in CSVD-CI	Raw mean±SD	Primary evidence	Small sample; null result.	Main alpha-diversity table; https://doi.org/10.1177/13872877261418554

Table S5B. Beta-diversity and community-composition summary

ID	Report	Beta-diversity/community-composition finding
1	**Fongang et al., 2023**	Continuous imaging analysis; no case-control beta-diversity comparison.
2	**Saji et al., 2019**	T-RFLP/enterotype approach; no sequencing-derived beta-diversity analysis.
3	**Cai et al., 2026**	Jaccard-based community differences mainly between aCSVD and aCSVD-iNPH; alpha-diversity group differences were not significant.
4	**Xu et al., 2026**	Bray–Curtis community composition differed across HC, WMH without cognitive impairment, and WMH with cognitive impairment (PERMANOVA $p = 0.025$).
5	**Song et al., 2024**	Multi-omics community differences were reported; exact effect metrics were not consistently available.
6	**Huang et al., 2024**	Bray–Curtis community composition differed between aCSVD and controls (adonis $R^2 = 0.08$, $p = 0.001$).
7	**Zhang et al., 2024**	Ordination and group comparisons were reported; this report overlaps the preferred Anhui alpha-diversity report.
8	**Shi et al., 2023**	PLS-DA/ordination was descriptive; no conventional inferential beta-diversity test was retained.
9	**Cai et al., 2021**	PLS-DA/related displays were descriptive; no conventional inferential beta-diversity effect was used.
10	**Saji et al., 2021**	T-RFLP/enterotype approach; no sequencing-derived beta-diversity analysis.
11	**Huang et al., 2024**	Alpha-diversity and MRI regression were the principal focus; overlapping Anhui cohort.
12	**Chen et al., 2026**	PLS-DA was descriptive; no conventional inferential beta-diversity effect was used.

Table S6. Taxon–MRI/CSVD association and taxonomic hierarchy map

The original reported taxon label and taxonomic rank are preserved. Multiplicity-corrected, nominal, and unadjusted findings are distinguished in the Model/correction and Key caution columns.

Study	Reported taxon	Reported rank/unit	Phylum	Class	Order	Family	Genus	Species	MRI/CSVD phenotype	Reported association	Interpretive direction	Statistic	Model/correction	Key caution
Shi et al., 2023	*Collinsella*	genus	Actinomycetota	Coriobacteriia	Coriobacteriales	Coriobacteriaceae	***Collinsella***		Total and periventricular Fazekas scores	Higher abundance associated with higher scores	Unfavourable	Positive correlations reported in the unadjusted correlation matrix	Unadjusted	No clear matrix-wide correction.
Song et al., 2024	*Coprococcus*	genus	Bacillota	Clostridia	Eubacteriales	Lachnospiraceae	***Coprococcus***		Severe-WMH CSVD versus controls	Lower in CSVD/severe-WMH phenotype	Favourable	Differential abundance	Group comparison	Potential cross-cohort concordance.
Fongang et al., 2023	*Pseudobutyrivibrio*	genus	Bacillota	Clostridia	Eubacteriales	Lachnospiraceae	***Pseudobutyrivibrio***		WMH volume and PSMD	Higher abundance associated with lower burden	Favourable	$p < 0.001$ in abstract-level summary	Multivariable; BH correction	Population-based continuous outcome.
Huang et al., 2024	*Roseburia intestinalis*	species	Bacillota	Clostridia	Eubacteriales	Lachnospiraceae	*Roseburia*	***Roseburia intestinalis***	MRI-defined aCSVD versus controls	Lower in aCSVD	Favourable	Differential abundance	BH-adjusted species-level differential-abundance testing	No lesion-specific MRI association.
Xu et al., 2026	*Acetivibrio*	genus	Bacillota	Clostridia	Eubacteriales	Oscillospiraceae	***Acetivibrio***		ALPS index / free-water pathway and cognition	Associated with MoCA through metabolite and ALPS mediation	Pathway-specific	Indirect effects reported in mediation models	Cross-sectional mediation	Not causal.
Huang et al., 2024	*Faecalibacterium prausnitzii*	species	Bacillota	Clostridia	Eubacteriales	Oscillospiraceae	*Faecalibacterium*	***Faecalibacterium prausnitzii***	MRI-defined aCSVD versus controls	Lower in aCSVD	Favourable	Differential abundance	BH-adjusted species-level differential-abundance testing	No lesion-specific MRI association.
Fongang et al., 2023	*Ruminococcus*	genus	Bacillota	Clostridia	Eubacteriales	Oscillospiraceae	***Ruminococcus***		WMH, PSMD and executive function	Higher abundance associated with lower WMH and PSMD and better executive function	Favourable	$p < 0.001$ for WMH/PSMD; $p < 0.01$ for executive function in report summary	Multivariable; BH correction	Population-based continuous outcomes.
Cai et al., 2026	*Solobacterium*	genus	Bacillota	Erysipelotrichia	Erysipelotrichales	Erysipelotrichaceae	***Solobacterium***		Basal-ganglia and deep CMB counts	Higher abundance associated with fewer CMBs	Favourable	$r = -0.292$ and -0.296; FDR $p = 0.019$ and 0.018	Spearman; selected FDR	aCSVD-iNPH subgroup; denominator ambiguity.
Shi et al., 2023	*Anaeroglobus*	genus	Bacillota	Negativicutes	Veillonellales	Veillonellaceae	***Anaeroglobus***		CSVD versus controls	Higher in CSVD	Unfavourable	FDR-significant differential abundance	FDR-corrected group comparison	Not a direct MRI correlation.
Zhang et al., 2024	*Dialister*	genus	Bacillota	Negativicutes	Veillonellales	Veillonellaceae	***Dialister***		CBF/mfALFF and CBF/mReHo neurovascular-coupling indices	Higher abundance associated with lower NVC indices	Favourable	Survived multiplicity correction; exact coefficients in supplement	Adjusted correlation	Anhui cohort overlap.
Shi et al., 2023	*Megasphaera*	genus	Bacillota	Negativicutes	Veillonellales	Veillonellaceae	***Megasphaera***		CSVD versus controls	Nominally higher in CSVD	Unfavourable	Did not survive FDR correction	Nominal group comparison	Opposite direction; nominal only.
Song et al., 2024	*Megasphaera*	genus	Bacillota	Negativicutes	Veillonellales	Veillonellaceae	***Megasphaera***		Severe-WMH CSVD versus controls	Lower in CSVD	Favourable	Differential abundance	Group comparison	Potential Anhui overlap.
Shi et al., 2023	*Veillonella*	genus	Bacillota	Negativicutes	Veillonellales	Veillonellaceae	***Veillonella***		Lacune count	Higher abundance associated with more lacunes	Unfavourable	Positive correlation reported in the unadjusted correlation matrix	Unadjusted	No clear matrix-wide correction.
Saji et al., 2021	***Bacteroides***	enterotype / genus-dominant community	Bacteroidota	Bacteroidia	Bacteroidales	Bacteroidaceae	*Bacteroides*		WMH presence	Enterotype I associated with higher WMH odds	Unfavourable	Fully adjusted OR 3.2 (95% CI 0.98–11.47), $p = 0.06$	Age, sex, education, vascular risks	Borderline fully adjusted estimate; overlaps Saji 2019.
Fongang et al., 2023	*Barnesiella intestinihominis*	species	Bacteroidota	Bacteroidia	Bacteroidales	Barnesiellaceae	*Barnesiella*	***Barnesiella intestinihominis***	WMH volume and PSMD / overall CSVD imaging burden	Lower abundance associated with higher burden	Favourable	q/p values reported in the supplement; source article contains internally inconsistent directional wording	Multivariable; BH correction	Source reports inconsistent direction; interpret cautiously.

Study	Reported taxon	Reported rank/unit	Phylum	Class	Order	Family	Genus	Species	MRI/CSVD phenotype	Reported association	Interpretive direction	Statistic	Model/correction	Key caution
Song et al., 2024	*Barnesiella*	genus	Bacteroidota	Bacteroidia	Bacteroidales	Barnesiellaceae	***Barnesiella***		Severe-WMH CSVD versus controls	Lower in CSVD/severe-WMH phenotype	Favourable	Differential abundance	Group comparison	Not a continuous MRI association.
Chen et al., 2026	***Prevotella-9***	genus-cluster	Bacteroidota	Bacteroidia	Bacteroidales	Prevotellaceae	*Prevotella-9* (16S database cluster)		CSVD with cognitive impairment versus controls	Lower in CSVD-CI	Favourable	Differential abundance	Small case-control study	16S database cluster; not a formal species.
Cai et al., 2026	*Thermus*	genus	Deinococcota	Deinococci	Thermales	Thermaceae	***Thermus***		Deep medullary vein score	Higher abundance associated with higher DMV score	Unfavourable	$r = 0.271$; FDR $p = 0.031$	Spearman; selected FDR	Clinical direction unclear; possible environmental/contamination concern.
Chen et al., 2026	*Fusobacterium*	genus	Fusobacteriota	Fusobacteriia	Fusobacteriales	Fusobacteriaceae	***Fusobacterium***		CSVD with cognitive impairment versus controls	Higher in CSVD-CI	Unfavourable	Differential abundance	Small case-control study	Small sample; descriptive.
Shi et al., 2023	*Parasutterella*	genus	Pseudomonadota	Betaproteobacteria	Burkholderiales	Sutterellaceae	***Parasutterella***		CSVD versus controls	Higher in CSVD	Unfavourable	FDR-significant differential abundance	FDR-corrected group comparison	Not a direct MRI correlation.
Cai et al., 2021	Enterobacteriaceae	family / related lineage	Pseudomonadota	Gammaproteobacteria	Enterobacterales	**Enterobacteriaceae**			Total CSVD burden	Higher abundance associated with greater burden	Unfavourable	Exploratory burden association	Largely unadjusted	Exact taxon label varies within the lineage.
Song et al., 2024	*Haemophilus*	genus	Pseudomonadota	Gammaproteobacteria	Pasteurellales	Pasteurellaceae	***Haemophilus***		Multimodal MRI measures via metabolite associations	Taxon–metabolite–imaging chain reported	Pathway-specific	Specific coefficients in supplement	Correlation network; FDR varies	Severe-WMH Anhui; possible overlap.
Shi et al., 2023	*Pseudomonas*	genus	Pseudomonadota	Gammaproteobacteria	Pseudomonadales	Pseudomonadaceae	***Pseudomonas***		CSVD versus controls	Lower in CSVD (LEfSe)	Favourable	LEfSe result	Differential abundance	Opposite direction across studies.
Xu et al., 2026	*Pseudomonas*	genus	Pseudomonadota	Gammaproteobacteria	Pseudomonadales	Pseudomonadaceae	***Pseudomonas***		WMH cognitive subgroups	Higher in WMH-CI in reported pattern	Unfavourable	Group comparison	Post-hoc correction status unclear for this specific comparison	Potential overlap.
Cai et al., 2021	Proteobacteria	phylum	**Pseudomonadota (published label: Proteobacteria)**						WMH burden	Higher abundance associated with greater WMH	Unfavourable	Exploratory correlation	Exploratory; no clear matrix-wide multiplicity correction	Broad phylum; source-specific nomenclature.
Huang et al., 2024	Proteobacteria	phylum	**Pseudomonadota (published label: Proteobacteria)**						MRI-defined aCSVD versus controls	Higher in aCSVD	Unfavourable	Differential abundance	Case-control shotgun analysis	Phylum-level ecological signal.
Song et al., 2024	*Akkermansia*	genus	Verrucomicrobiota	Verrucomicrobiae	Verrucomicrobiales	Akkermansiaceae	***Akkermansia***		Multimodal MRI measures via metabolite associations	Taxon–metabolite–imaging chain reported	Pathway-specific	Specific coefficients in supplement	Correlation network; FDR varies	Not direct replication of disease enrichment.

Table S7. JBI appraisal of included analytical cross-sectional reports

The JBI analytical cross-sectional tool was applied to the target result in each report. Judgments were recorded as Yes, No, Unclear, or Not applicable; no numerical summary score was calculated. For result-level items, judgments refer to the target result listed in Table S7A.

Table S7A. Item-level appraisal of target results

ID	Report	Target result	Q1	Q2	Q3	Q4	Q5	Q6	Q7	Q8
1	Fongang 2023	*Barnesiella* / taxon associations with WMH, PSMD and EF	Yes	Yes	Yes	Yes	Yes	Yes	Yes	Yes
2	Saji 2019	Enterotype I vs WMH within GIMLET related report	Yes	Yes	Yes	Yes	Yes	No	Yes	Yes
3	Cai 2026	*Solobacterium*/*Thermus* correlations with CMB/DMV	Yes	Yes	Yes	Yes	Yes	No	Yes	Yes
4	Xu 2026	WMH-related microbiome differences and microbiome–metabolite–glymphatic/cognition associations	Yes	Yes	Yes	Yes	Yes	Yes	Yes	Yes
5	Song 2024	Differential gut microbiota in severe WMH-predominant CSVD and downstream multi-omics/neuroimaging associations	Yes	Yes	Yes	Yes	Yes	Yes	Yes	Yes
6	Huang 2024 multi-omics	aCSVD vs HC diversity/taxonomic differences and functional metagenomic/metabolomic findings	Yes	Unclear	Yes	Unclear	Yes	No	Yes	Yes
7	Zhang 2024	*Dialister* / alpha diversity associations with neurovascular coupling and cognition	Yes	Yes	Yes	Yes	Yes	Yes	Yes	Yes
8	Shi 2023	*Collinsella*–Fazekas and *Veillonella*–lacune associations	Yes	Yes	Yes	Yes	Yes	No	Yes	Yes
9	Cai 2021	Proteobacteria/Enterobacterales-related associations with WMH and total CSVD burden	Yes	Yes	Yes	Yes	Yes	No	Yes	Yes
10	Saji 2021	*Bacteroides*-dominant enterotype I → WMH, fully adjusted odds ratio	Yes	Yes	Yes	Yes	Yes	Yes	Yes	Yes
11	Huang 2024 alpha	ln(Chao1) → CMB count and modified total CSVD burden	Yes	Yes	Yes	Yes	Yes	Yes	Yes	Yes
12	Chen 2026	Alpha-diversity case–control comparison and microbiome–UMRR associations	Yes	Unclear	Unclear	Yes	Yes	No	Yes	Yes

JBI questions: Q1, Were the criteria for inclusion in the sample clearly defined?; Q2, Were objective, standard criteria used for measurement of the condition?; Q3, Was the exposure measured in a valid and reliable way?; Q4, Were the outcomes measured in a valid and reliable way?; Q5, Were confounding factors identified?; Q6, Were strategies to deal with confounding factors stated?; Q7, Was appropriate statistical analysis used?; Q8, Were the study subjects and the setting described in detail?

Table S7B. Methodological considerations relevant to interpretation

ID	Report	Key methodological consideration	Consequence for synthesis
1	Fongang 2023	Large population-based sample with objective imaging, multivariable adjustment, and Benjamini–Hochberg correction. Some secondary taxon directions are inconsistently reported.	Use adjusted taxon–MRI findings as outcome-specific evidence; do not treat individual taxa as replicated biomarkers.
2	Saji 2019	Crude enterotype–WMH comparison; same GIMLET cohort family as Saji 2021.	Use only unique information and do not count as an independent replication of Saji 2021.
3	Cai 2026	FDR-corrected taxon–CMB/DMV correlations; no vascular-risk adjustment.	Treat as exploratory, result-specific evidence.
4	Xu 2026	Modern 16S/multi-omics and MRI phenotyping with covariate adjustment; mediation is cross-sectional and the cohort probably overlaps other Anhui reports.	Use as hypothesis-generating multi-omics/glymphatic evidence; avoid double counting the Anhui family.
5	Song 2024	Severe-WMH phenotype with matching and sensitivity analyses; selected phenotype and probable Anhui overlap limit generalizability and independence.	Use narratively; do not count as an independent Anhui replication.
6	Huang 2024 multi-omics	Shotgun metagenomics is strong, but aCSVD criteria/MRI burden reporting are incomplete and main group comparisons were not vascular-risk adjusted.	Interpret functional findings narratively and case-control contrasts cautiously.
7	Zhang 2024	Detailed microbiome/MRI methods with adjusted partial correlations and multiplicity correction. WMH volume differed across subgroups but was not included in the principal microbiome–NVC models; the cohort is related to other Anhui reports.	Use for unique neurovascular-coupling outcomes; avoid duplicate counting within the Anhui family.
8	Shi 2023	Structural MRI–taxon correlations were unadjusted; matrix-wide correction was not clearly reported.	Treat the MRI–taxon associations as exploratory narrative findings.
9	Cai 2021	MRI burden used standard criteria, but microbiome–MRI correlations were unadjusted; the paper also contains a Spearman/Pearson reporting inconsistency.	Use imaging associations narratively; keep human observational and ex vivo evidence separate.
10	Saji 2021	The WMH model was extensively adjusted, but only 23 events were present and the estimate was imprecise; the sample overlaps Saji 2019.	Retain the adjusted WMH estimate but do not count it independently from Saji 2019.
11	Huang 2024 alpha	Adjusted Chao1–MRI models; modified 0–8 burden score, a source-labelled OR from a count model, and a sample nested within the Anhui family.	Use as the preferred alpha-diversity–structural MRI report; interpret the CMB estimate as an exponentiated count-model effect.
12	Chen 2026	CSVD-CI criteria and the human sequencing protocol are incompletely reported; hypertension differed between groups, analyses were unadjusted, and the sample was very small.	Use alpha-diversity and permeability findings narratively; keep human observational and mouse FMT evidence separate.

Supplementary abbreviations

aCSVD, arteriosclerotic cerebral small vessel disease; aCSVD-iNPH, arteriosclerotic cerebral small vessel disease with idiopathic normal-pressure hydrocephalus; ALFF, amplitude of low-frequency fluctuation; ALPS index, index of diffusivity along the perivascular spaces; ASV, amplicon sequence variant; BH, Benjamini–Hochberg; BMI, body mass index; CBF, cerebral blood flow; CI, confidence interval; CMB, cerebral microbleed; CSVD, cerebral small vessel disease; CSVD-CI, cerebral small vessel disease with cognitive impairment; CSVD-MCI, cerebral small vessel disease with mild cognitive impairment; CSVD-NCI, cerebral small vessel disease without cognitive impairment; DMV, deep medullary vein; EPVS, enlarged perivascular spaces; F/B, Firmicutes/Bacteroidetes ratio; FDR, false discovery rate; FMT, fecal microbiota transplantation; HC, healthy control; iNPH, idiopathic normal-pressure hydrocephalus; JBI, Joanna Briggs Institute; LEfSe, linear discriminant analysis effect size; MCI, mild cognitive impairment; MRI, magnetic resonance imaging; NCI, no cognitive impairment; NVC, neurovascular coupling; OR, odds ratio; OTU, operational taxonomic unit; PCoA, principal coordinates analysis; PERMANOVA, permutational multivariate analysis of variance; PLS-DA, partial least-squares discriminant analysis; PSMD, peak width of skeletonized mean diffusivity; PVS, perivascular spaces; SLI, silent lacunar infarct; SVD, small vessel disease; T-RFLP, terminal restriction fragment length polymorphism; UMRR, urinary mannitol recovery rate; WMH, white matter hyperintensity; WMH-CI, white matter hyperintensities with cognitive impairment; WMH-NC, white matter hyperintensities with normal cognition; WMHV, white matter hyperintensity volume.

Reference numbers used in the cohort-overlap table correspond to the main manuscript reference list.
